# Treatment of Criminal Lunatics;—The Case of Captain Johnston

**Published:** 1853-01-01

**Authors:** 


### Art. II.—TREATMENT OF CRIMINAL LUNATICS;—THE CASE OF CAPTAIN JOHNSTON.

Never before the present period has the spirit of progress been more active in efforts to revolutionize and change the social and political condition of this country. The old do-nothing principle has been gradually yielding to innovations, which, once, would have been condemned as dangerous to society and tending to the utter destruction of our venerable, time-honoured institutions; until, at this present moment, there is left scarcely one portion of that fabric we were so proud of a century ago, which has not undergone the sifting process, and been either totally or politically changed. In no other institution has this progress towards improvement made such rapid strides as in the administration of the law; the commencement of which may be traced back to the period when the penal code was modified; and it is greatly to be desired that, in our present zeal for change, the same spirit of humanity and Christian philanthropy which characterized that most important and admirable innovation, will advance in its holy work of amelioration, until our whole system of criminal punishment has become so improved that the finger of the most captious objector cannot point, with reason, to one defect. While we rejoice to acknowledge that much has been accomplished in the direction of rational innovation on our antiquated penal system, we have to regret that the law which governs our practice in cases of criminal lunatics, has received so little attention, and is permitted to remain like a foul, disgraceful stain upon the humanity, justice, and good sense of the nation.

We do not affirm that the criminal who escapes the legal punishment annexed to his crime, on the plea of insanity, is unjustly treated, when, for his own safety and the protection of society, he is placed under restraint; neither do we hold that this necessary restraint should be relaxed, while he continues under the influence of disease: but we urge that it is not humane to treat a man so situated, whatever may have been his crime, with harshness and severity; and it is far from just, to continue any restraint beyond the period of his recovery. At present, the condition of a criminal lunatic is a painful exception to our mild, equitable, well-adjusted judicial rule for the correction of offenders. He stands in an anomalous position; for, though he is acknowledged to be irresponsible for his actions, and consequently innocent of premeditative guilt, he is treated with a harshness which only a sane and wilful act of profligacy, or vice, would justify. Instead of being regarded as the unfortunate victim of the heaviest misfortune which can fall upon the head of man, a misfortune to which all are exposed, a condition which deprives him of all self-control, and reduces him to the rank of a machine acted upon by mere animal instincts and passions;—instead of that kind consideration of his state, which so great a calamity has the right to demand from humanity;—instead of placing him in the most favourable situation, and bringing to bear upon him all the appliances which our present improved science offers for his recovery;—the poor, diseased, irresponsible lunatic is treated with a severity of punishment which far exceeds any, not excepting even death, to which the worst of criminals are subjected. Is this an exaggerated description of his treatment, does any one suppose? Then let him obtain an order to visit the criminal lunatic's ward in Bethlem Hospital; there he will see that, so far from the picture being too highly coloured, it falls far short of the actual condition of those unfortunate men whom he will behold mingled together, in one long, dark, dismal corridor; the better with the worse, the mild with the malicious, the timid with the violent, the mere mono-maniac with the most furious madman,—with no classification, or separate treatment, proportioned to their varied conditions. Is this calculated to promote recovery? Is this in accordance with our improved views of mental disease, and present rational methods of cure? Far from it; but this is not the worst part of the case. Under the present condition of the criminal lunatic, perhaps it is a happy circumstance that his chances of restoration to sanity are so small; for recovery would bring to the unfortunate prisoner no relaxation of his punishment, and must only add to his wretchedness, by increasing his sense of the degradation and misery of his state. Will it be credited by the humane reader, that the incarceration of the criminal lunatic is perpetual! and that after recovery, though he is, in theory, an innocent man, one not to be held responsible for acts committed during the existence of his disease, he is to be kept a prisoner during her Majesty's pleasure, and therefore may be subjected to all the ignominy and harshness, and even danger, of the criminal ward for the whole period of life. The punishment—for we cannot use a milder term—of the criminal lunatic who has recovered his senses, is one which far exceeds that to which any other description of offender is subjected. Some felons are imprisoned at home, and others are banished to the penal settlements; but from none is the hope of a mitigation or abridgment of captivity taken. There is always this to sustain the man under the heaviest privations; he knows that good conduct will have its usual effect in moderating his sufferings, and finally removing them. But from even this consolation the poor lunatic, who in a moment of irresponsible frenzy has committed a criminal act, is excluded. There is no liberation for him; and only to death can he look for the termination of his heavy sufferings.

Now, when we reflect, apart from the consideration of the question of justice or injustice of any particular case, that every man is liable to the attacks of insanity—and all, under the influence of this disease, may be impelled to the commission of crimes which will subject the criminal to the punishment of the Bethlem ward,—it behoves us all to exert our influence to remove this stigma from our penal statute-book.

From the commission of wilful and sane acts of criminality, we endeavour to guard ourselves by the inculcation of moral and religious sentiments in our systems of educational training, and, under the blessing of God, we may reasonably hope that this effort to subdue or keep under our natural instincts and passions will be so effectual, that we may pass through life with every expectation of escaping the dreadful consequences of crime; but what will preserve us from insanity and its consequences? What prophylactic remedies, what moral or physical preventives can we adopt, that will keep at bay the horrible calamity to whose invasion we are exposed? Is any man exempt? Have we not seen the highest order of intellect subdued by this disease, and the best trained mind, morally and intellectually, compelled to succumb to its potent influence? Then, if we are all liable to mental disease, none can tell that he will not, under the influence of impulsive passion, uncontrolled rage, or some fancy of his perturbed imagination, commit offences, from the mere thought of which he would, if sane, shrink with horror and disgust; but which may bring upon him the dreadful punishment of perpetual incarceration in the dismal ward of a lunatic asylum, where he must associate with men labouring under every degree of mental disease, and stained with every shade of immorality and crime!

We have been led into this train of thought at the present moment by the perusal of a pamphlet written by Captain Johnston, late of the ship *Tory*, at this moment a prisoner in Bethlem Hospital on the charge of murder committed on board his ship during a paroxysm of insanity. A powerful effort was made at the time of his trial to fix upon him the capital offence, but from this he escaped on the plea of insanity. According to the usual custom in such cases, the unfortunate man was then placed under restraint, in which he has continued for a period of nearly seven years, the greater part of which time he has been, according to his own statement, and the *certificate of the physician of the hospital, Sir A. Morrison*,* in a healthy condition of mind, and perfectly conscious of his dreadful situation.

We do not pretend to affirm from our own personal knowledge the correctness of this opinion, though we confess that we see in his statement presumptive proof of restored reason. It is a plain, connected narrative of facts, the tendency of which is to show, that the criminal acts committed by the writer were the consequence of a sudden paroxysm of insanity, induced by long-continued mental anxiety and bodily fatigue, occasioned by the mutinous conduct of his crew. We will now give this extraordinary narrative *in extenso*, with no alterations, except the omission of names—for obvious reasons—premising only that the statement of facts here submitted to the reader was written wholly by Captain Johnston, without a single document to aid him, or any friendly co-operation. It was, moreover, written four years after the occurrences narrated, and corresponds exactly in the minutest particulars with the statement made by him at the time of his trial.

### CAPTAIN JOHNSTON'S STATEMENT.

On the 24th of January, 1844, I sailed from Liverpool, in command of the ship *Tory*, East Indiaman, of 600 tons, bound on a voyage to Bombay, freighted with a general cargo, and consigned to Messrs.

* "Mrs. Johnston,

"Madam,—I am favoured with yours of the 16th instant, respecting your husband, Captain Johnston. A quarterly report is regularly made to the Secretary of State for the Home Department, of the state of mind of the inmates of the criminal department of Bethlem Hospital, in which that of Captain Johnston is included. I may add that Captain Johnston has never exhibited any symptoms of insanity since he was placed under my care. "I remain, Madam, your obedient servant,

"Alexr. Morrison."

Forbes and Co., gentlemen whose memory I shall ever cherish for their past kindness. It behoves me to speak in grateful, but not familiar terms of them, as I happen to be one of the many who have shared their beneficence.

At 7 P.M. I discharged the pilot and the steamer which had me in tow, and stood to the N.W., under all sail, with a faint breeze from W.S.W., approaching almost to a calm, and continued variable, with light airs during the latter part of the night—daylight, a fresh gale from the westward, causing every rope and yard of canvas to bear equal strain, and lasted till 5 P.M. the same night: it then veered its course round to the northward, blowing a pleasant gale from that quarter, which carried me clear of the land, gradually sinking Old England and those I loved beneath the horizon. The breeze blew strong and steadily in that direction until I reached within the limits of the N.E. trades. The commencement of the voyage was in every respect calculated to ensure success, so far as wind and weather were concerned: the ship was also staunch and true, and in a fair state of discipline, such as to afford company and harmony to every man on board, but the more especially to the individual whose character was solely depending on it. At this time fortune seemed to favour me from all points of the compass. I was happy, and tried to distribute it to those by whom I was surrounded, joyfully speculating on the future as I journeyed on to the land of my troubles. About the 14th of February I crossed the equator; I had a pleasant run up to the meridian of the Cape—from thence to Bombay. The voyage proved tedious, having experienced a great deal of light winds and calms—however, it was good on the whole. On the 15th of May, I saw the high land to the southward of Bombay, being the first after sailing nearly 15,000 miles between contrary winds and currents. When I arrived at Bombay the cholera was raging violently, which carried off several of the crew: some of them were sent to the hospital; five solicited their discharge, which I granted—not a usual thing in a foreign port, when the wages are up at a high rate, as they then were, owing to the sickness among the shipping. I shipped others in their stead, and sailed on the 26th of June for Canton, freighted with a cargo of cotton and opium, and addressed to Messrs. Dent and Co. I had a prosperous run round to China, calling at Singapore, giving the crew a short respite, in order to refresh them. On the 1st of August I came to an anchor in Canton River, and remained there for nearly three months, in the expectation of obtaining a charter for England, but was unable to get it, in consequence of a number of small ships lying at that port, and the remarkably low freights: I therefore proceeded to Singapore, where I succeeded in obtaining a cargo of Straits' produce for Shanghae in China. On the 28th of November I sailed from thence, and proceeded through the Caramata passage, Java Sea Pits, and Dampair Straits, where I encountered many light, baffling winds and calms. After getting clear of these Straits and into the Pacific Ocean, I experienced very heavy weather: I shall ever remember the time, on account of an accident which proved fatal to a young man, whilst he was performing his duty. Several days before it occurred the weather was rough and squally, so very peculiar to tropical latitudes, especially along the north coast of New Guinea.

One of these squalls was rapidly approaching the ship, and orders were given to take in the top-gallant sails. This young man happened to be the first on the yard; but unfortunately the sail caught him in the face, and pitched him head foremost on the quarter deck. In his fall his forehead came in contact with the front of the poop, which knocked the scalp clean off—the most deplorable sight I ever beheld. The poor fellow seemed insensible to pain, till the scalp was assisted to its former position; he then called out in the most agonizing tone. He was immediately removed to the cabin, where he soon expired. Poor fellow—the last words he uttered were, his "Poor mother," and requested to have the Lord's Prayer read to him. He had received a liberal education, and must have belonged to respectable parents; but I could never find out where they resided. He told me a few days before his death, that he had been in the capacity of clerk in a merchant's office, before he came to sea. I shipped him in Singapore; two days previous to my departure from that island, I took him into the half deck, on account of his respectable behaviour. During the time which I commanded that ship, I never allowed a respectable boy to mingle with the crew, knowing the demoralising effects which their corrupt morals have on a young mind.

On the 25th of January, 1845, I arrived off Wosung, and took the ground in standing in to the anchorage. On the 9th of February I moored ship off Shanghae, where I remained for some time. I think it was about the 1st of March, that a mutiny broke out on board of the barque, *Charles Jones*, belonging to Liverpool. In this serious affair, the chief-mate was nearly murdered by the crew, and would have fallen a victim to their murderous intent, had it not been for the kind exertions of my (then) chief-mate, and two or three of my crew, who went on board, and rendered speedy and important services in terminating the fray. My chief-mate was so closely pressed on one occasion, that he was compelled to jump overboard to save his life. Her crew then got possession of the ship, but did not hold their station long. My mate got on board, and they were again defeated; but he received some heavy blows before it was settled. Her chief-mate was severely injured, having two of his fingers broken, and his hand much bruised. It appears from the evidence placed before H. B. Majesty's Consulate of that port, to have occurred in the following manner:—The crew got access to the cargo stowed in the forehold, and whilst they were in the act of embezzling it, the mate detected them, ordered them out of the hold, and desired them to put on the hatches. They refused the order, and commenced maltreating him. This caused the disturbance, and I believe, led on by the second mate, (as he was charged as one of the number.) The master having no officer to assist him in carrying on the ship's duty, and to oblige him in his unfortunate situation, I parted with one of my officers, little thinking that my own sad calamity was so fast approaching, and that a greater loss was to take place amongst my ship's company, in the next port where I sailed to. On the 5th of April I sailed from thence, and arrived at Hong Kong on the 14th. After making the necessary arrangements with my consignees, I sailed on the 21st for Whampoa. Whilst lying there, four seamen belonging to Her Majesty's steam ship, *Driver*, committed a most atrocious murder in presence of my ship's company, and according to the depositions taken down in my cabin, it occurred as follows:—These men got liberty from their commanding officer to go on shore, and they immediately proceeded to the individual's boat, on board of which he resided, following the occupation of a baker, employed in that capacity by an European residing at Hong Kong. As soon as they went on board of his boat, they demanded spirits from him, which he very liberally granted, until he thought that they had had sufficient; finding that they could get no more, they became violent, and abused him; seeing that his situation was rather a critical one, and that reason would have no effect or influence upon them, he jumped into his little boat, telling them of his intention to go on board of the steam ship and report them. They pursued him with all speed, and just as he was in the act of passing the *Tory's* stern, making direct to the steamer, they caught him, and dragged him into their own boat—took their handkerchiefs from around their necks—bound him hand and foot—and pitched him into the river, calling out to my ship's company to look at them, and they would show them how to serve the —— baker. I have related this unfortunate occurrence, because it was partly owing to it that my own unfortunate circumstances were brought about.

On the 9th of May I sailed from Whampoa, and called at Hong Kong, to receive on board the remainder of my cargo, in accordance with charter party. When I arrived in this Island, the Government subpœnaed my chief-mate, a petty officer, and a seaman, to appear as evidence against the four seamen already mentioned; I was therefore obliged to ship the first that presented themselves. Independent of this unforeseen circumstance, I was compelled to give a passage to England to two women under false characters, which tended greatly in bringing on the ills which befell me. All these things took place precisely as I was on the point of weighing anchor. When I sailed, the change of the monsoons was setting in, and every preparation was making for a typhoon, which necessarily made me more anxious to leave the port, with scarcely an officer to assist me—it would have been better if I had had no officer at all. The man whom I shipped as chief-mate was not an hour on board before he destroyed a boat, in presence of a young man, in Messrs. Dent and Co.'s employ, who came on board to see me off; and the last words he said, descending the ship's side, were, "Good bye, Johnston; I am afraid that you are to have an unpleasant voyage, if I may judge from what I have already seen." I did not proceed far without experiencing something similar, but of a more serious nature, through his entire disobedience to orders. Two days previous to the setting in of the S.W. monsoon, the weather became unsettled and threatening, shewing indications of some approaching storm, during which time it required my presence on deck, to await the results, which were hourly expected, and which generally take place in this quarter of the globe, without giving much warning, particularly about the change. I think it was about the 21st, then in latitude 17° North, and longitude 113° East, or nearly on the meridian of Canton, that a fresh gale sprung up from the southward. Canvas was reduced to double-reefed topsails. At four p.m. the gale seemed to blow steadily. I retired to my cabin at an early hour, to get a little rest after the fatigue of the two nights' watching, leaving strict orders with the chief-mate to call me at the slightest appearance of any change, telling him to be sure and give me timely notice should it commence to rain, knowing that rain would be certain signs in bringing on a sudden shift of the wind. The orders were completely neglected—the gale had moderated—the rain had fallen in torrents—and I received no intimation of it. About ten p.m. the ship was taken aback: I had the greatest difficulty in reaching the deck, the ship having been hove over almost on her beam ends with the violence of the squall. After giving my instructions to the man at the wheel, I then endeavoured to let go the ropes belonging to the after sails; by this time several ropes had parted aloft, and the foresail split, which soon eased the ship. It was proved afterwards that the watch had taken refuge in the forecastle, to shelter themselves from the rain; consequently, when their services were required, at that critical moment they could not be found. The same night, I think, there was a total eclipse of the moon, which brought on dreadful weather, blowing from various points of the compass with great violence, causing the sea to rise in pyramids, forcing the ship to labour, twist, and strain heavily, and make large quantities of water, the sea breaking over her fore and aft. I had some difficulty in getting the pumps attended to; a part of the crew were taken sick with a fever, which happens very frequently to many of the skulking fellows, who pass ashore as fine open-hearted jolly Jack Tars. I don't mean to insinuate that they are all the same; what I mean to say is this—that three out of every five are in reality nothing but mere impostors on ship board, conspiring mischief, and proclaiming themselves lawless; they not only disturb their own peace of mind, but they make it their constant study and delight in sacrificing the happiness of those whom they mingle with; and when they are in a foreign land, they go roving about in a continued state of drunkenness, knocking down and shamefully abusing the natives, wherever they may be. I had the misfortune to have many of these renegades on board, which can be proved by respectable parties residing at Canton, with reference to a portion of my crew. One man was fined by the British Consul at that port, for maltreating the Chinese, and attempting to murder one of them; and would most undoubtedly have succeeded, were it not for the interference of a gentleman named Forbes, the American Consul. My family are in possession of these documents, written by Messrs. Dent and Co., who paid the fine. The same man was imprisoned at Singapore for similar offences. He could not be trusted out of the ship, without something serious being brought to his charge. He was a desperate fellow, and a worthless seaman—incompetent to steer the ship, if she exceeded five miles an hour in her rate.

My instructions were to beat down the China Seas, in order to shorten the voyage; but seeing no possibility of carrying my design into execution, owing to the bad quality of my crew, I therefore bore up for the Northumberland Straits, adopting the eastern route. After I got clear of these Straits, and into fine weather, I found that my health began to fail me, having been so long exposed to the inclemency of the late gales. I had been standing forty-eight hours in wet clothes, without an opportunity of changing my dress, in consequence of which I was taken with pains all over my body, which brought on a palpitation of the heart, causing the most excruciating pain, that I could scarcely draw my breath. It was the second time that I had been visited by the same complaint. The first time occurred on a former voyage, caused by exposure to the night air, and want of my natural rest; this happened in 1841, on my outward-bound voyage to Bombay. I had a Dr. Cruikshank on board at the time, who relieved me by proper depletion, and strong medicine. I was now placed beyond the reach of medical aid, and under the necessity of having recourse to the only expedient, venesection, and no one but myself to perform the operation. After bleeding freely I found relief. I remained in a weak state for nearly three weeks, only going on deck to take my observations, to ascertain the true position of the ship. During my debility a quarrel took place between R—— and S——. S—— was laid up with the venereal disease, the third time since our leaving England. He became quite filthy with dirt and vermin, so much so, that the stench and effluvia from his person were too noxious to withstand; he likewise became insolent. R—— came flying into my cabin, in a perfect rage, about S——, saying, "If he *was* an owner's man, that he would take —— good care not to take any of his —— slack." I told him that I did not allow anybody on board to give insolence to their superiors, and if he had done so, I should expel him from the quarter-deck altogether, and gave orders to turn him into the forecastle. S—— finding that he had lost the good things, felt very much mortified, and instead of feeling grateful for the kindness he had received up to this date, he swore to have revenge before the ship should reach her destination: and he stood true to his word. He now began his machinations, and soon found means to keep the pot boiling, and combined with the two licentious women. Had the ship been manned with devils from the regions of the damned, she could not have been in a worse state of insubordination than she was at the time I speak of. My situation was a precarious one, and I used every precaution within my power; but she still resembled a demonomy.

About the 10th of July, running under a heavy press of sail in Pitt's Passage, with the wind on the larboard quarter, I wished to alter the ship's course, to clear a detached shoal, and gave orders to have it done as expeditiously as possible; in which case, the man C—— became very insolent, and tried to obstruct the others—preventing the ship's duty from going forward—and let go the swinging boom guy by the run. He then walked to where several of his party were standing, in conversation with two women, and called out, "Now is the time, my boys,"

these harlots cheering them on. He was the most obstructive, perverse, and ill-natured fellow I ever came in contact with. They were not all of one mind at this time. On the 15th I came to an anchor at Lambuck, where I sent R—— on shore, in charge of the boat, to purchase live stock, and other necessary stores, handing him 129 rupees, and giving him strict injunctions not to allow the boat's crew to get intoxicated when they landed. I did not deem it prudent to leave the ship, she being in the unsettled state which I have represented.

When the boat returned, the greater part of them were under the influence of drink: the man J—— in a beastly state of intoxication, that he was obliged to be hoisted on board like a dog. Two bags of rice and four bags of sweet potatoes were damaged: three goats and about a dozen-and-a-half of fowls were drowned. In coming alongside, R—— called out to the men in the boat to keep themselves steady, and don't let the —— see that you have been drinking, a constant expression of his, and one of his mildest terms. I took no notice of it—J—— was carried into the forecastle, where he went to sleep. After remaining there about two hours, he came staggering aft to where I was walking, and addressed me in these words:—"Captain Johnston, I am sorry for you, sir, I know of something which concerns you, but I shall not tell you what it is just now—there are some rum fellows on board of this ship, but I don't care a d—n for them." I desired him to walk forward, and told him to go to sleep. Shortly after, I saw T. R—— stripping off his clothes, and challenging F—— out to fight him; I immediately sent M—— to prevent them striking each other; however, this did not stop them from exchanging blows. 16th.—At daylight the boat was sent on shore with all the empty water-casks to get filled. At noon, the water was towed alongside on a raft, hoisted on board, and discharged into the tank, and the boat started off the second time. When she returned in the afternoon (5 p.m.), there were two hands missing. I inquired of R—— the cause of their disappearance: he said they had run away from the boat. I asked if they had their clothes with them: he told me they had not. I then sent M—— to ascertain the truth from the sick men, who were laid up in the forecastle; they informed him that the two men carried two bundles out of their berths a few minutes before the boats shoved off. R—— knew of it, but denied it. I dispatched the boat with information to the head man of the island to apprehend them and send them on board. He returned an answer, saying, "It was impossible for him to capture them, as they had taken themselves to the woods." 17th.—I got under weigh, having on board 28 men, all told, and 2,200 gallons of water, a quantity which ought to have lasted eighty days at least, if it had been properly distributed to the crew, allowing each man seven pints per day. R—— took charge of the water; but as he was short of clothes, he was under the necessity of washing his things in fresh water, consuming large quantities for that purpose. It could not be expected that I should attend to all these things myself. My duty was to navigate the ship: his duty to see no waste nor embezzlement committed in the ship under his especial charge. Fresh provisions were served out to the people for five days after I sailed from the last-named island. Vegetable food was not all expended for nine weeks afterwards. About the latter part of July, the trades were blowing strong and squally. In one of these squalls the mizen topsail split, and orders were given to bend another. Whilst the people were getting the new sail to pass, I observed R—— striking an old man in the face and over the head, a fine helmsman and a harmless fellow—indeed he was the only individual out of her complement of tars who could steer the ship in a seaman-like style. I ordered the mate to desist, and told him that his conduct was ignominious and unofficer-like. He flew into a fury, and poured forth such torrents of blasphemy never heard during the whole course of my life:—he swore by the Holy Ghost, by the Holy Trinity, and the calendar of all the saints. Not long after, he was standing on the top-gallant forecastle head, dealing out some of his most refined sentences, when, lo and behold! the lower studding sail sheet parted, and knocked him down senseless on the spot where he stood. He was carried into the cabin in a state of insensibility—leeches were applied to the injured part, and all other remedies were put in practice for his restoration;—he remained in his cabin for two weeks, during which time everything went on in a quiet and orderly manner. I remarked to him, a day or two before he got perfectly well, that the crew would never respect him if he did not refrain from using such profane language. He said, "My last captain used to preach the like sermons, but he gave it up for a bad job." I have often given a —— myself when things were going on left-handed, but never carried it to extremes. His general speech was quite unbecoming an officer. He soon took charge of his watch again.

By this time I was approaching the meridian of Cape St. Mary, and the weather beginning to get unsettled, I came on deck one night, and found the ship running two points off her course, and not an officer looking out or upon the deck, although she was under a heavy press of sail; before the hands were mustered and canvass reduced, the squall had struck the ship, blowing to pieces some of the small sails,—the mizen topsail-yard was likewise carried away, and all through his carelessness. In a few days after this occurred, I went on deck about 5 a.m., and found the quarter-deck again deserted by the officer in charge. I walked towards the man at the wheel, and saw him bleeding profusely at the nose and face, the same person whom R—— abused on a former occasion. I asked him by whom did he receive the injury; he told me that the mate had taken a spite at him ever since I had interfered in his behalf, and had called him a —— foreign ——, and that he was the captain's fancy-man. I called one of the boys who was standing in front of the poop, and desired him to find the mate, and tell him that I desired to speak to him. He came aft, and I took him into my cabin, where I reasoned with him regarding his ill-treatment to the old man. I also gave him to understand that his behaviour was too revolting to be much longer tolerated, and reminded him of a circumstance respecting his late ship. I then begged of him to be more circumspect in future, and not to let me hear such complaints as those brought against him, as I was held responsible for all his actions. Two days before I saw the south coast of Africa, the ship going large, carrying starboard studding sails fore and aft, low and aloft, I gave orders to R—— to set the ringtail, but previous to doing so to reeve new halyards, the old ones being worn out. He set the sail without paying any attention to the latter request, and when the sheet was hauled aft, the halyards parted, and away went the sail: it was towed to pieces, the ship having great speed through the water: this was rather annoying, and helped to prolong the voyage, as also the loss of property. To a landsman it may appear trifling, to me it was of some consequence: when there are things destroyed by accident, or act of God, there is no blame to be attached to any officer; but when an officer acts contrary to all rules, and in defiance to all orders, it certainly becomes unpleasant to the individual who must render an account for the losses sustained, and whose character depends on the result of his voyage: I was doing everything in my power to shorten it; he was working in opposition to me—I was anxious for the general good of all parties connected with ship and cargo. I had lost sails, split sails, sprung spars, carried away spars,—there was more damage done in the short space of time that he was on board, than happened to me from the time I took charge of the ship. I remonstrated with the man in private; and told him, if he would not act in conformity with my commands, that I should be under the necessity of dispensing with his services altogether; and then said, "Now, Mr. R——, I hope there will be no need of my calling you to question until the voyage is terminated." The first expression which he uttered when he returned to his duty, and within hearing of the people, "I don't care a d—n for his —— cuddy court martial." I took care that it never came to a cuddy court martial again. His mean spirit now prompted him to pursue a different course:—he leagued in with the most desperate characters, and endeavoured to rouse their feelings against me. From this time I never could find him on the quarter-deck when he had charge of the watch; he kept continually forward among the crew. I have very often found the ship running two or three points out of her course, and all the yards out of trim, and the studding sails beating to pieces. To the westward of Cape Recy I encountered stormy weather.

In one of these storms it blew with dreadful violence, which obliged me to take in all sail of the ship, and was nearly losing them all before the task was accomplished. The ship lay quite with her side buried in the water, till the waves began to rise, and the gale abate. She then required after sail, to prevent her from falling off in the trough of the sea. In clewing up the main topsail, at the commencement of the gale, one of the reef earrings got jammed in the larboard main topsail brace block. I had given orders to clear it, as I could not work the yards so long as it remained in that state. Five hours had elapsed, and nothing done in the way of clearing it. I asked the officers the cause of its not having been righted; they said that they had tried to relieve it, but could not succeed. I told them they ought to have sent up a preventive brace, and cut the standing part of the other, and sent the block on deck. They found some excuse. I was compelled to go aloft, and take a pair of the maintop gallant studding sail halyards out of the maintop, reeve it through the spare-block at the main top-mast head, carry them out to the main topsail yardarm, and bend them on to the double part of the earring which jammed the block. I did this in presence of my ship's company, and not one of them offered to assist me, although every indulgence had been shown to them. They were allowed 2 lbs. of beef, 1 pint of rice, 1 lb. of bread per day, on Sunday, Monday, Wednesday, and Saturday; $1\frac{1}{2}$ lb. of pork, and pease soup, and the same quantity of bread, on Tuesday, Thursday, and Friday, and flour pudding three times a-week. I also allowed them watch and watch in cold, or very warm weather; hot coffee and a glass of brandy every time that the watch came on deck in a stormy night, and a glass of grog whenever they reefed top-sails. There were no spirits on the ship's articles. It was in this hurricane that I supposed my chronometers had altered their rate. When I made the south coast of Africa, they were quite correct.

The man J—— was hove over the wheel—I carried him into my cabin in my own arms, and waited on him till he recovered.

About ten or twelve days after, I rounded the Cape, with a strong gale at S. E., and shaped my course for the island of St. Helena N. W. by N. $\frac{3}{4}$ N., altering it occasionally, as circumstances required. I think it was about the 14th of September, and at 10 p.m., that I gave orders to shorten sail, having run my estimated distance, according to my position at noon. The studding sails were all taken in, royals and top gallant sails were stowed, the courses hauled up, and the ship rounded too, with her head to the eastward, under the three topsails, jib, and spanker, having experienced a strong westerly set from latitude 29°. Daylight—the weather a little squally, with small rain, which hindered me from seeing any great distance. I ascertained the course and distance of the ship, from the time she was hauled to the wind, which placed me within fifteen miles of the land. If the weather had been clear, I should have seen the land, at least sixty miles off. I would have given my right arm at the time for the sight of that island, for many reasons. I was charged with passing it intentionally. My latitude by the sun's meridian altitude placed me precisely on the parallel of it. I called the mate in presence of the ship's company, and spoke to him thus:—"Mr. R——, something has happened to my chronometers, and not long since; I have never found them to deceive me before, and since rounding the Cape I have had no opportunity of obtaining the longitude by any other method, only time-keepers. You are aware that I have always embraced every chance of the kind, whenever it presented itself. I think that I am about forty miles to the westward; I am very sorry for it; however, it can't be helped. You say that there is three feet of water in the tank, which will be sufficient to last us fourteen days longer. Square away the yards, and set all sail;" and accordingly I shaped my course for the

Island of Ascension. On the following morning I made further inquiries respecting the quantity of water, and found that there was not above half the quantity which R—— reported. I then ordered all hands to be put on an allowance of a quart per man per day. There was much going and coming amongst the crew, and the general clamour was, "He intends to pass all the islands, and run home without water." I could never find out the origination of the falsehood; it was evidently got up in order to excite the crew to revolt, making this a pretext for their motives. The next day they all struck, and refused to do any more duty. R—— brought me the report. I went and addressed them in these words, "I find that you are all labouring under some erroneous and foul-founded impression. You have denied your duty, because of an accident over which I had no control; and you seem determined to take the advantage of it by mutinous measures. I strongly advise you to return to your respective duties. I assure you it will be far better for you. I also tell you that I hold my life in as high esteem as you hold yours. Recollect what I have told you—all that I desire of you is, to steer the ship." These were all the words which I exchanged with the crew. D—— in his evidence said I threatened to hang them up to the mainstay, like porpoises, and make them drink each other's blood.

After I retired to my cabin, R—— came to me and said, "Captain Johnston, if I had been in your place, I would have shot every man of them who would deny their duty. I sailed with a Captain D——, belonging to New York, who would very soon do it if they had acted the same to him as they have done to you. You are too easy with them; they will soon be your master." I told him that I should act as I thought proper in these matters, and that he might depend upon it that it would give them or any one else a little trouble before they became my master; and said, "I perfectly comprehend you, Mr. R——; I hope you will understand me." This was all that passed between us, there were two of the boys present at the time.

About the 19th, and at 2·30 a.m., I came on deck and asked R—— if he saw the land, he answered me in the negative, and was insolent in his deportment towards me. In a few minutes I observed the crew mustering in front of the poop; I then inquired the meaning of the crew standing there: he told me that he was expecting some rain, and that he had brought them aft to save water. Something struck me at the time from his manner, he appeared much confused: I immediately went and dressed myself, and returned upon deck, being the last time that I had undressed or slept on board of that ship till she was moored in the West India Dock, London. Fortune now began to dispute my claims on her—she thought that she had done enough for me; so I was left to my own resources. My perplexities increased, and my troubles augmented daily. I gave orders to take in all the studding sails, and brought the ship to, with her head to the eastward. At daylight the weather was gloomy, with light squalls and small rain. I stood on the same tack, close hauled, till noon—

the sun obscure. I then wore the ship to the westward, and steered due west under all sail, keeping in the parallel, thinking I might be to the eastward. The carpenter went several times to the mast-head to look for the land: none of the others ever attempted to go aloft, although they had called me by all sorts of opprobrious epithets and names calculated to affect and strain the temper. 20th—At 2 a.m. tacked ship to the eastward, and stood on that tack till noon—the sun was again obscure. I then called R—— and M——, and consulted with them on the best method to be adopted, leaving it entirely to their own suggestions. I told them to be candid, and let me have their opinion on the subject. They both said they were at a loss, and wished to know what I proposed myself. I pointed out the two courses which lay before me, stating—"If I shape my course to Pernambuco, it will take eight or nine days before we can reach that port, and if we proceed on the voyage, we shall be apt to fall in with some outward-bound merchantman, who will give us a supply of water, and that will be nothing more than what I have done to others in similar circumstances." They both said that my proposal was the best and most prudent. I said, "Well, my dear fellows, recollect that you have sanctioned it at your own free will, so let us have no after-thoughts. I find that the crew have got villanous intentions from their behaviour: men can never disguise their feelings when they are of a criminal nature. I have been keeping a strict watch lately; I am no stranger nor greenhorn on board ship—I can see further than people imagine. If there be any foul attempts made at my life, I shall sell it as dear as I can. There is another thing I request of you, you are not to allow S. C——, T. R——, L——, nor D—— to take the wheel, nor come upon the quarter-deck until you receive my orders. You will go and lay the yards square, set all the studding sails, the ship's course is N. by E. ½ E.; you will find that they will refuse to make sail, but I must have it done, and the sooner the better. We are only losing time, and can never find the Rock in the midst of the ocean, though we are close to it." I then dispatched the tidings to the crew, telling them that I did not require them to do any duty, only to steer the ship. No man could have acted more reasonably or rationally. When the orders were given to set the sails, three of them stepped out from their berths, when C—— said "The first —— that puts a hand to the halyards, I will lodge this knife in the —— heart." I called out "Never mind the vagabond, if he dares to put his villanous threat in execution, I shall comb his hair with a pistol-ball,—look smart and set the sails, and when you have done it spread the awnings, and let the people remain under them out of the sun." After these things were completed I went to my cabin, loaded two pistols, and discharged them through the stern windows as a signal, to show them that I was making preparations to defend myself. In the evening, the two women were trying everything in their power to excite the men to revolt. I desired R—— to go forward and separate them from the ship's company, and turn them below out of the way. He refused, and I addressed him in these words—"Mr. R——, it appears very strange to me that the crew take such liberties with you when you are performing your duty—there is something wrong—it will come out by-and-by." Next morning I found C—— at the wheel: I called the mate and said, "Mr. R——, did I not tell you not to allow C—— at the wheel, he can't steer the ship, and I shall not permit him to remain there—I will have my orders obeyed—I am the responsible person, and I must be the imperative man." The following morning at daylight a sail in sight, but at too great a distance to signalize with him, and bearing dead in the wind's eye, his topsails dipping in the horizon. This was the first day I observed M—— insolent in his behaviour towards me. I said, "Mr. M——, it is a pity that that ship did not pass to the leeward of us." He made no reply, but looked as black as a thunder cloud. I took little notice, only returning my looks with that contempt which his merited. I had done a great deal for him during the voyage, but he was unqualified to appreciate it. Sept. 24th. —A sail in sight, bearing about two points on the starboard bow, and standing to the southward; I immediately gave orders to take in all the studding sails, and steered direct for him. I then signalized with him; he proved to be the French barque ——, from ——, and bound to Calcutta. I asked him if he could supply me with a cask of water and a little fresh provisions, as I had some of my crew lying sick; he told me he should be most happy to oblige me as far as it lay in his power. I ordered the boat to be hoisted out, and took R—— and S—— into my cabin, and ordered the steward to get some of my clean clothes for them, to make them appear a little respectable going on board a strange ship. The boat went off, and was absent about two hours, returning with stores amounting in value to 760 francs, and amongst them there was a live pig,—the pig was killed for the use of the hands, one half for their dinners that day, and the remainder for the following day. When the boat returned, R—— came aft to where I was standing with the sextant in my hand, and said, "Captain Johnston, the French captain desired me to tell you that he had a cask of small wine to dispose of, such as he gave to his own ship's company instead of spirits; and, should you feel inclined to purchase it, he would let you have it at prime cost, 40 or 50f." I said, "Take it, of course, 'tis just the thing; and I dare say it will please our fellows as well as if it were of the best quality." The boat shoved off again, and was about an hour or so away; in rounding the ship's stern, my attention was attracted by the noise of the boat's crew—I looked over the ship's side, and saw that the boat's side was stove in; it was a splendid 14-oared cutter, ten months old, and capable of carrying all hands in case of an accident happening to the ship: she was built of teak, and copper-fastened—a better boat never belonged to my ship. I was remarkably fond of the boat, and felt grieved to see it in such a shameful condition. I questioned R—— about it, when he flew into a passion, and said, "—— the —— boat, I could not carry her on my back." This was the first time I had checked him in the presence of the ship's company, which made him show off his independent notions. "No, R——," said I, "you are unable to carry the boat, efficient officers have a very different method of doing their duty, and would have paid attention to the property placed under their charge, more particularly alongside of a stranger." He then talked about jumping overboard, when I said I wished he had never jumped inboard. I ordered the boat to be hoisted up, and stowed on the starboard side of the poop to undergo repair. The yards were again trimmed, and the ship went her course as before; I gave orders to stop their allowance of spirits, as I could perceive they had had sufficient for one day. I obtained five different sets of distances from the sun and moon, which placed me thirty-seven miles to the eastward of my chronometers, being the first chance of the kind since doubling the Cape: the moon was in distance, and in her last quarter. Captain Horseburgh, in his East India Directory, describes the same similitude of weather in his voyages from the Cape; he says that he has run from thence to England without having one single instance of determining the longitude by lunar observation. Decks being cleared, and all things fairly arranged, I retired to my cabin; S—— was seated there, unable to do duty. I said, "Well, S——, how did you like the Frenchman?" He answered, "Very well indeed, sir." "I dare say," said I, "they treated you with all their national civility; I think the captain is a gentlemanly fellow, judging from the style of his letter—did you drink anything on board of him?" "Yes, sir, I had a cup of coffee and a little brandy in it." "I think the mate has had some too," said I. "Yes, he had some, and likewise all of the boat's crew got a glass of grog—they also drank a quantity of small wine, which the French sailors served out to them, and they also broached the cask after we shoved off from him." Nothing took place till 8 30 p. m., I was then seated in my cabin, the officers had just gone on deck, after taking their eight o'clock grog; they were not above a quarter of an hour absent, when B. Y—— walked into my cabin, and addressed me thus—"Captain Johnston, has the mate informed you of the crew's intention against your life?" "No, B——, he has not, but I am, nevertheless, aware that there is something very dark and mysterious going on amongst them. Are you certain R—— knows of it?" "I heard them tell him," said he. "If he knows I have told you, I am sure he will take away my life; he has placed the greatest reliance in me, and says he is sure I can never betray his confidence." "If this be the case, how could you expect him to acquaint me, if you are one of his accomplices and swore fidelity to him." "I never swore to him; I have given you the information in order to put you on your guard, considering it my duty by so doing: you have been a good master to me, and treated me more like a father than the master of a ship. Don't you remember the night that you expected to make the Island of Ascension? the mate was forward with the people all the time that you were below, and when you came on deck, before you gave orders to take in sail, they followed him aft; and when I observed you walking towards the ship's side, I made sure their intentions were to pitch you overboard; but when I saw you face about, I was not so much afraid. One or two proposed to heave you in the sea when you were in the act of taking your observations, and off your guard; and another suggested putting you in the coal-hole, and starving you to death." My blood began to boil at the conclusion of the last sentence, and I went on as follows:—"B——, tell me the names of the men who are the most interested in this infernal conspiracy and diabolical torture." He then pointed out these, saying they were the principal leaders—"S. C——, T. R——, L——, D——, and G——." "Can you swear to it?" "I can." "Is there any one besides you who knows it?" "Yes, sir; and ready to substantiate the charge to their faces. I heard F—— tell M—— to look out for yourself: the mates are acting very deceitful to you—whenever you are below, they are continually with the men; and after you have given orders to pipe to dinner, they call out to the men, 'A horse's dinner—no water with it;' and when they have done trimming the yards, they say it is done to keep them moving; there was no occasion for it; and all kind of disagreeable things, to enrage the feelings of the people."—"Do you know what were the communications which F—— imparted to M——?" "Yes, sir; he told them that the crew were determined to take your life, and run the ship to America!"—"B——, will you fight for me, if it be required?" "I will, till I die."—"Inform Mr. R——, M——, S——, S——, and the steward, that I wish to speak with them." The boys came first, M—— and S—— followed, R—— came last of all, his countenance changing colour; not a word was spoken on either side—all was silence, till he made his appearance. I broke silence in these words:—"Well, Mr. R——, I have just received intelligence of a damnable conspiracy going forward, aimed at my life, and the intent of making off with the property placed under my charge—I have already told you that it would come to light ere long. You are all aware that I have done everything in my power to make you all comfortable and happy; I have felt a deal of care and anxiety on your account; I have not had an hour's sleep in comfort since I came on the parallel of St. Helena; I would have much rather lost an arm than have passed those islands without seeing them." Every commander of a ship must acknowledge that nothing could be so mortifying to a man's feelings as to have it said by his ship's company that he is unable to find the islands in question, although it has frequently happened to many navigators. "Are any of you aware of the people's intentions towards me? B—— has this moment told me that they have determined on killing me; it is the first time the boy has spoken to me during the voyage; and he also says that there are several persons in the ship who are ready to affirm it." S—— was the first that spoke, and these were his words:—"I have seen a great deal of secresy going forward lately, and I have been thinking that there must be mischief at the bottom of it." "Well, carpenter, the top and bottom is nothing more nor less than the taking of my life by torture; but before I relinquish it to these sons of Neptune, I would prefer taking it myself. Carpenter, get the irons, I shall secure the ringleaders till I fall in with some ship, otherwise I will call at the Cape de Verds, and in all probability there will be some English cruiser on that station, or close to its vicinity, I might be apt fall in with one in a day or two." The carpenter and the boy B—— then armed themselves; I gave two pistols to the mate, but took care that neither of them were charged with ball, in case of getting the contents lodged in my own body. The boy B—— had also told me that the portion of the crew he had pointed out by name, had likewise signed papers the day before I fell in with the French barque, and R—— and M—— were acquainted of its reality. I then went on deck, and desired them to follow me. When I reached there, I ordered M—— to pipe all hands to muster, and said, "Now recollect, the first man that refuses to do his duty, I shall look upon as one of their party, and treat him accordingly." The crew were then mustered—some of them came forward directly—the main part hung back and began to mutter something between themselves. I called out to fall in here, all of you; I want to ask you a reasonable question: you are at liberty to answer me as you please. When they were all collected, I put the following questions to them:—"Have I ever treated you unkindly? Have I ever called you out of your names? Have I not behaved to you with kindness and humanity? Have I not waited on the sick? Have I not carried the sick into my own cabin? Have I not given my own allowance of water to the sick, when my own wants were sore and hard to bear? What motives can you assign to me for passing the island? Do you suppose that I have done so from private reasons or intentionally? How could I navigate the ship without your assistance? Is not my allowance of water served out the same as another man's? The water has been wasted, and I have received false information respecting it." Ten or twelve called out "Yes, sir,—yes, sir, 'tis true; you have behaved well to us." I told B—— to point out those men who were to take my life, and who had purposed the basest torture for its accomplishment. He called them by name:—C—— was the first, L—— the second, R—— the third. "Carpenter," said I, "secure these men in irons, and walk forward the rest of you." D—— then requested to speak with me: I told him I would shortly. R—— ran forward; I desired the officers to go and bring the man aft—none of them would obey the order. I followed him myself, and said, "R——, if you do not submit to be put in irons, I shall lodge the contents of this pistol in your body." He had placed himself at the bowsprit end; I went after him, caught him by the collar, and struck him over the back several times with the flat of my sword, and conveyed him to the spot where the others were placed: the carpenter secured him. I then addressed the three men in these words—"Oh, you damnable and cowardly villains!—and you intended to cut me in pieces—how would you like to be served so yourselves?" I pointed the sword to their breast and said, "I have a good mind to run you through," and gave each of them a tap over the shoulders with the flat of the weapon, and gave orders to have them separated from the rest of the ship's company, and placed where they could have no chance of communicating with each other; and said, "R——, if these men get adrift, I shall look to you as one connected with them." He replied, "I shall take —— good care of that." C—— spoke, and said, "Don't be too fast, R——."

I did not stab the men in irons, as it was represented and sworn to; I made use of threats such as I have stated, and brandished the weapon before them in order to terrify them. My position justified my proceedings: I not only had a right to secure these men, but I had also the power of shooting the man who dared to refuse to aid me in securing them. I had experienced ample proofs of their villanous designs, and the strongest demonstrations of their mutinous proceedings; they had very often obstructed me in the performance of my duty, and as often denied theirs. I was beset by a lot of unprincipled and desperate characters, who felt no compunction for their atrocious crimes, whom no reason could reconcile, and whose rebellious spirits were carried beyond the extreme limits of endurance; I was likewise situated far beyond the realm, and surrounded by the foaming elements without an officer to support my authority; neither could I appeal to a magistrate for assistance—no going out at the back door and bringing in the help at the front one. If I had had proper and efficient officers on board of that ship, there would have been no cause for me to change a muscle in my face.

When these things were settled, D—— came to me and asked my pardon, and said that he would tell me everything concerning it if I would pledge my word to pardon him, and not enter him in the log-book as one of the mutineers; he then detailed the whole of their intended plans, coinciding exactly with the boy B——'s account of the affair. I ordered the prisoners to be sent in the tops—R—— in the fore, L—— in the main, C—— in the mizzen, the latter being accused as the leading man—he did not deny it when I interrogated him on the matter. I addressed R—— in these words—" R——, this serves me right; I ought to have turned you out of the ship in Whampoa for your conduct there; but your solicitations prevailed, and you are now only waiting an opportunity of becoming my murderer, although you have repeatedly acknowledged there and elsewhere, that you were never so happy in any ship." I offered this man his discharge in the above-named port, but I could not get clear of him. I sent for J——, T——, B——, and S——; they seemed most favourable to my cause: I asked them if they had any hand in the plot; they told me they had not. " Why," I demanded, " did you not give me notice when you were aware of it, if you objected to become one of their party?" " We thought," said they, " that you had been apprized of it, and were expecting this examination every hour, having made sure that F—— had reported all that was going on."—" I don't believe," said I, " that you would be guilty of any such wickedness. I wish you, J—— and T——, to be under arms in your watch on deck; B—— and S—— will relieve you when their watch comes on deck—just walk in front of the poop, and give me notice of any occurrence taking place." I felt confident of their fidelity, because they expressed their sincerity in the mildest and strongest terms. I gave them two muskets mounted with bayonets, and desired them to be faithful to me, and not to allow the weapons out of their hands. After these things were arranged, I went on deck; the woman B—— was standing in the steerage hatch; R—— flew at her, and said, "Oh, —— —— will take your life!" he then knocked her down, and a squabble commenced between them below; two or three fellows descended the hatchway, and entered into conversation; the noise then subsided. In about ten minutes they came on deck; when I ordered them to march forward. Thus ends the first night.

25th. At daylight I gave orders to set the starboard maintop gallant and main-royal studding sails before all, they being set abaft all, and the trades inclining to the larboard quarter, causing these sails to shake at intervals.

I then retired to my cabin, telling R—— to keep a sharp look-out for shipping, and acquaint me should any heave in sight. B. Y—— belonged to the first mate's watch, and he was called on by that officer to go aloft, and perform the task which I have just mentioned. When the boy reached the maintop gallant yard arm, he called out to lower away the royal studding sail. R—— ordered those on deck to take hold of the sheet, and drag the son of a —— overboard—that's the —— that split. 10.30 a sail hove in sight, which proved to be an American whaler, bound on a whaling voyage to the South Pacific—the trades light and variable, ship making little progress through the water. After the sun passed the meridian, the trades increased to a gentle breeze, and I soon came up with the stranger. Sent the boat on board of him, with an empty water-cask—R—— in charge. It was getting on for 4 p.m. when she returned with a full cask; it was hoisted on board, likewise the boat. Yards were again trimmed, and I proceeded on my voyage as usual. After the decks were cleared away, and things set in order for the night, Y—— related what had transpired between him and the mate in the morning. He said, "Captain Johnson, that man will have your life—you will not believe me—ask F——, if you doubt my word, he heard what he said; and when you told him to bring you the papers out of the forecastle, which the men had signed, he hove them overboard, and informed you that he could not find them." I sent for M——, and desired him to send F—— aft, to whom I put these questions—"I understand that you were the first man who gave notice of the schemes against my life?" F——, "Yes, sir; I am the first man who made your officers acquainted with the facts relative to their designs which you are now in possession of; but it looked very strange to me, that you seemed to take no notice of it yourself." I then said, "The mates kept it in perfect obscurity from me." F——, "That was the reason why I told B—— to tell you all the particulars connected with it from beginning to end. They are all trying to make friends with you now, in professing their entire ignorance, seeing your determination to obstruct its farther progression. They are all aware of the plot of taking your life, and running the ship to the continent. I now leave my life under your protection, and I am ready to stand before your officers and crew, and maintain it to their faces." Thus ends F——'s account. I went on deck—M—— had charge of the watch. I told him to hand me the pistol I had desired him to arm himself with last night; he said it was lying by the man at the wheel. "Indeed!" said I, "that is a very fine spot for you to place the weapon in: come below, I wish to speak with you. Tell Mr. R—— to accompany you. Carpenter, look out for the ship's steerage, until you receive further orders." When they made their appearance, I said—Mr. M——, is it true that F—— gave you warning, how the people intended to massacre me, and run off with the ship, the same as I stated last night?" His reply was, "Yes, sir; he did."—"How often did he tell you so?" "Twice."—"You never gave me the least intimation of it." "I reported it to my senior officer; I thought it my duty to do so."—"No, sir; you don't know your duty, or you would have told me (when you saw the thing was not conveyed to me) the instant you communicated the tidings to him. Have you any hand in this vile confederacy yourself?" "No, Captain Johnston; I have nothing to do with it." "M——, (said I,) if I thought you a party concerned, I should think very little of running you through." I felt very sorry to bring such a charge against my officers, and put it off till it became too pressing. I then addressed myself to R——. "Mr. R——, what have you to say in your defence?" "Nothing, Captain Johnston; I beg your pardon. Forgive me this time, and I shall never do the like again. I am a married man; oh! forgive me." I said, "You talk of being a married man, and just about becoming a murderer, in the most cool and deliberate manner. Am I not a married man? and do not I love my wife? Yes! you false fellow." He then fell on his knees, and asked forgiveness. I desired him to get up from that posture, and face me like a man; and said, "Will these boys pardon you? I am not afraid of you individually, so long as I can keep my eye on yours." S—— and G—— both spoke, and said, "No, Captain Johnston; he will kill you, and then he will finish us." G—— also stated that R—— had threatened his life, only a few days ago, in these words: "I will serve you out shortly, you Scotch son of a ——." "You hear what the boys say, R——; they are terrified of their persons, if I permit you to go at large." I dismissed M—— and F——, and told them to send S—— below. When he came, I said, "Well, carpenter, it has all come to light at last. R—— has acknowledged their beautiful combination against me, whilst they are wearing my clothes on their backs." S—— said, "I thought as much; these men have not looked me straight in the face for the last five days. I have also observed them separate whenever they saw me walking to where they were standing in conversation with each other; and which has all been delivered in whispers, and black looks. S—— was sitting in my cabin, and heard it all; often repeating these words, 'Oh, my God! Captain Johnston, they will have your life, and afterwards take mine.' R—— swore he would heave me overboard a little time before I fell from the main stay." But, although S—— spoke in this manner, I had very strong suspicions that he was one of their confederates, having been so closely connected with its first origin. He was unable to do duty of the slightest description, and preferred associating with the most furious and designing portion of the crew. The greatest blackguard became his confidential and bosom friend; and with these characters he was able to unburden his passions, and discuss his lewdness, to their admiration. I found it an utter impossibility to keep him from holding these mysterious interviews, during the disturbance; he must either have been casting off his former alliance in amicable terms, or otherwise entering into a new treaty, in order to carry out his preconcerted threats of revenge. No man in the universe could surpass him in cunning and artfulness. No man in the world could have worked out the total destruction of his fellow-man more scientifically than he did mine. At the time I was addressing myself to S——, R—— disappeared, and in the course of ten minutes after he vanished, I heard the sound of many feet approaching the cabin door. I desired the carpenter to go and ascertain the meaning of this new disorder. I then ordered the boy to hand me two pistols and the sword. I snapped one of the pistols at the entrance of the companion door, and followed its flash to deck. They all fled instantly—they were all armed with large bolts and capstan bars. The two individuals who were stationed there, and had pledged themselves to stand faithful to the last, had retreated with their comrades. I caught J—— by the collar, as he was making off, and spoke to him thus:—"You —— cowardly fellow, you promised to stand true to me." He replied, "Oh, sir! they all came upon me in a moment, which made me afraid to sing out, for fear they would take my life." I called him a plausible villain. He then said, "That R—— threatened to run him through if he dared to speak; and caught the musket out of T——'s hand, and directed him to stand on the larboard side of the cabin door, and he would stand by the starboard side, and run you through with the bayonets as soon as you made your appearance, that the others were all crowded around, so I felt dreadfully afraid to call out. Oh, sir! I am all in a tremble!" I then pursued the main body. Some of them took to the rigging—several heavy bolts were discharged at me from the tops. I returned to the poop, and ordered S—— and F—— to go in search of R——. M—— had also deserted the quarter deck. They were about an hour hunting after R——, when they returned and told me they had found him, but he refused to accompany them. I said, "You must bring him to me; you cannot be blind to your real position." S—— said, "No, sir; we can see ourselves now—seeing is believing. I would shoot every man of them if I were in your situation; they will have your life, and mine too, if you don't use prompt measures. I could never have believed the thing, had I not been an eye-witness; after the man asking forgiveness, and promising to avoid them altogether." The man B—— came next, and said that R—— dragged him out of his bed, and ordered him to follow; saying, "Let us kill the —— at once; it is as good to be hung for a sheep as a lamb." T—— said that R—— went aloft when he left the cabin, and tried to cast the men adrift. He then told us they were coming to his aid, and insisted upon us to join him. S. C—— cheered the fellows when they retreated before me. I went up to the mizzen rigging to make sure whether the irons were taken off or not; and desired C—— to keep quiet, if he had any respect for himself. I gave him two or three slaps with the flat of the sword, and descended the rigging. I did not stab the man, nor beat him, as the evidence gave it against me.

After reaching the deck, R—— was brought to me by F—— and S——. F—— said that he wanted to jump overboard in the wash deck tub. I then put this question to him, "Well, R——, have you got anything to say for yourself?" He made no reply. "R——, you are a treacherous, base, and cowardly vagabond—your treachery exceeds everything." I struck him a blow with the butt end of the pistol, and ordered the carpenter to put the manacles on him. I never lifted a hand to the man before nor after. He was then removed to the long boat's stern. His party, seeing their leader defeated, began to crawl and cringe aft one by one, uttering these words :—"Captain Johnston, it is all his fault; he forced us aft, and told us that he would give us better times." The man C—— called out, "Yes, Captain Johnston, and run the ship to America, (that's what he told us,) if we lent a hand to kill you." I ordered the carpenter to bring the man aft, and let him hear these charges face to face. I was standing with my back against the mizzen rigging larboard side, and in the course of three minutes the carpenter sung out, "Catch him, catch him! he is adrift!" The man passed me like a shot, on the lee side of the quarter deck, ran abaft the starboard mizzen rigging, and sprung right into the sea, calling out lustily and hoarsely; some of the fellows swore that he gave three hurrahs. I sprang to the wheel, and hove it hard down, and gave orders to let fly the studding sail tacks, fore and aft. Not a soul among them would exert themselves to assist me in letting go a single rope. Every inch of canvas that could possibly draw had been set, so that the ship's rate was about seven miles and a half per hour. The sails were kept shaking in the wind for, I dare say, half an hour, and nothing done in the way of getting the boat out; one of the men singing out loudly, "Let the —— go." Another—"I hope the sharks have swallowed the —— up by this time." Another—"I hope the —— is in hell." Another—"The ship has been a hell afloat since he belonged to her." These were the expressions the men used when I was trying to get the boat hoisted into the water. These were the lamentations uttered by those individuals, who stood up in court, and gave their oaths that I drove the man overboard, after cutting and slashing at him for a length of time. Had the fellows persevered and exerted themselves like men, I might have stood some little chance of saving him. I know he was an excellent swimmer, at least he told me he could swim well. It being quite dark at the time, 4 a.m., 50 miles to the northward of the equator, this will show how far it was from daylight, the sun then vertical. The two women also gave their oaths that I followed the mate round the decks, cutting him severely, and that he was bleeding profusely all over the body. These creatures were under the hatches during the whole of the tumult. I took care to have that compartment secured, to stop the crew from having access there, because the carpenter's tools were in the steerage.

After the unfortunate man was gone, I was fully impressed that the crew would never have taken arms against me had he not forced them to it. I told them that I did not wish to hear anything more from them : I also told them if I had another recurrence of that kind, that I would sweep the decks fore and aft : whatever you have to say for yourselves relate it all to S——, he will take it down. Six or seven of them went into the cabin where S—— was, and gave their statement in their own words, and signed their names to it—I was quite ignorant of the contents, and never asked them for it. About ten a.m. in the same morning, the carpenter told me that the three men in the tops requested to speak with me. I asked him if he knew what they wanted; he said, "I think they intend to confess their villanies, and beseech your permission to return to their duty, finding it had all come to light." I said, "Carpenter, I don't think my life will be safe if I release them; however, I will hear what they have to say : keep yourself under arms, and send them to me." They came—C—— was the first, R—— the second, and L—— the third : C—— said, "Captain Johnston, we ask to be liberated out of irons, to beg your forgiveness, and to allow us to return to our duties,—we have acted very bad to you, sir,—you are the best man we ever sailed with,—you may thank your chief-mate for all the mischief which has happened on board of your ship,—he was the first who proposed killing you, and running the ship to America; we could never have thought of the like ourselves : oh! he was a —— rogue—he said that we could get into the back settlements of America, and would never be discovered there." They blamed the mate for all; he was gone, and they had it their own way. I listened to them till I got fairly sick of hearing them any longer : what they confessed to me was voluntary,—their statements were taken down and they signed it, not by compulsory measures, but entirely at their own option. I made use of no threats, nor attempts at violence; if I had been conscious of having acted wrong, or from the influence of drink it would have been my place and interest to have made it right with my ship's company by gentle persuasions and promises. How could I expect men to swear to a thing which I compelled them to acknowledge and sign at the point of the sword? These things are believed, but it is quite inconsistent with reason. Those men who appeared as witnesses against me swore that I stabbed the three men whilst they were in irons, and nothing was done to their wounds. It would be a very strange task for me to dress wounds which were never inflicted. The three men stood there without a mark or bruise on their bodies, with the exception of R—— ; one of his wrists was a little swollen, and a small portion of the skin broken, caused by his obstinacy while in the act of putting on the manacles. I spoke to them in these words—"If ever I find a repetition of this, you may rely on my word that you shall not get off so easily." I then dismissed them, and ordered them to their duty. C—— was the man that the boy B—— swore that I had beaten with my sword for the space of an hour, wounding him terribly, while in fact that man was going on with his shipmates, pulling and hauling at the braces, displaying more willingness and agility than he and they had done from the commencement of the voyage. The reader will not have forgotten the evidence on that occasion, where they stated that they never saw any symptoms of drink in me during the voyage up to the previous date, (24th.) Now the simple question I should wish to ask of any right-thinking man is—Could a man forget himself so far as to fly to drink and become a brute, after braving the trials and dangers of the voyage for them he lives for, and only six weeks' sail from their tender embraces? I demanded the log-book, and found nothing that had been entered from the 19th or 20th: this did not appear at all singular to me, but, on the contrary, strengthened the accounts as to R——. A ship's log-book is generally filled up at noon each day, unless there is something going forward very particular indeed,—this was the fatal day on which I gave it in charge of S——, he being the fittest to write the entries in the log: I then desired M—— and the boy B—— to remain with him, and write the occurrences just as they happened, and as they themselves saw it, bidding them not to enter anything but what they could swear to.

The night ended with light variable winds. I filled eight casks with rain-water: the crew on full allowance.—26th, at 4 p.m. the carpenter came running into my cabin in a great state of excitement. I asked the cause of his alarm. He replied, "Oh my God, Captain Johnston! one of the crew has just told me, that if it had not been for you I should have been a dead man last night. He showed me the knife, and said that that was to have entered my heart." I then asked him how they came to tell him these things. He said that they were relating all the instructions which R—— had given them. He (R——) had proposed to kill you first, and then had said that he would very soon do for the ——— Scotch carpenter. I said to him, "Well, S——, you must wear arms, and not allow them out of your hand while you are on deck, and do not let the men see that you are afraid of them; for if you show the least sign of fear, you may depend they will be sure to take the advantage of it. He then said, "Oh, sir! I feel terrified to go to sleep; whenever I try to shut my eyes, I start with fright." This was the intelligence S—— conveyed to me—the same S—— that stood up in the witness-box, and swore before heaven and earth that he saw no mutiny on board of the ship, except a little grumbling, and the crew walked forward at my bidding. Although he was well versed in deceit and ingratitude, his perjured soul will heave many heavy sighs before he is called upon to render up an account to his Eternal Judge; he was capable of selling soul and body for money, and ever ready for the highest bidder; he expected to have a haul out of me, but seeing no likelihood of its forthcoming, he then turned with all the vengeance and ferocity of a serpent. Shortly before my trial he often visited my family, lamenting his loss of time, and wondering who would remunerate him for his trouble.—On the 27th, the log was duly filled up, and signed by M——, S——, S——, B——, F——, and G——; it was then brought to me. I asked them if they had stated everything the same as it happened: they said "Precisely the same:" I did not affix my name to it until the following morning.

—28th. I fell in with the barque *Earl of Eglinton*, from Bombay, bound to Liverpool. At 3 30, I sent my boat on board of him with a letter, requesting the captain to pay me a visit in the morning; he sent me a reply, and solicited for a supply of wine, having passengers on board and beginning to run short of that article: I sent the boat back with some, and also a chest of tea.—29th. At 7 30 a.m. he came on board and breakfasted with me; we then compared chronometers, and according to his measurement of time in 11 days' run from the Island of St. Helena, where he had corrected his timepieces, it placed me 32 miles to the eastward of my chronometers, and 5 miles to the westward of my observations, 0. 2m. 8s. fast. I gave him the particulars of the mutiny, and acquainted him of my intention of calling at the Cape de Verds, thinking that there would be an English cruiser convenient to those islands. He said, (as was very true,) "that it could only be attended with loss of time and risk of losing the ship, should I fail in my hopes, as the navigation was very difficult for large ships:" this I knew from my own experience. He afterwards said, "If you feel apprehensive of your crew rising again, I will keep you company." I thanked him for his kindness, and told him "that I should not wish to be the cause of retarding him on his voyage." He spoke in the presence of my ship's company; and the men whom I was charged with cutting and wounding went on board of his ship, and remained on board for about two hours.

M—— had charge of the boat. S—— made one of the number, and wrote down the particulars of the mutiny on board of and in the log-book of that ship, in the presence of all the passengers. This vessel was two miles distant, and I on board of my own ship. If I forced him to make false entries in the *Tory's* log-book, it will surely be acknowledged that I had no power to compel him to give erroneous statements on board of a stranger when I was not there at all. The *Earl of Eglinton* arrived in Liverpool the same day that I sent the ship's letter-bag on shore off Plymouth, and was the first that reported the mutiny: we had parted company the same night that he boarded me, the *Tory's* draught of water being too much to bear him company in light winds, showing that, notwithstanding the many impediments which I encountered, the charge of drunkenness, and the three days lost by calling at the Island of Fayal, my mind and attention were devoted to the property under my charge. I forwarded a letter by the barque to my wife, written before I crossed the Equator, with the expectation of falling in with a faster ship, to report my progress; in this letter I wrote these words:—"I have had a mutiny on board since I commenced writing you." The same afternoon studding sails were set on both sides, the trades were blowing their last gasp, and I gave orders to take in the larboard lower studding sail—the halyards had got jammed in the jewel-block at the boom end—I called out to heave taut the halyards and send a man aloft with another pair, otherwise it would carry away the yard-arm; they paid no attention to the order, but kept pulling and hauling in defiance of my order, and away went the yard-arm close by the rigging. I could not help myself, not having a soul on board to enforce my commands: my situation was the most distressing of human calamities. My blood got inflamed, my eyes flashed fire, and everything around me appeared like the Aurora Borealis. I could not sleep—sometimes I would lie down on the carpet, with my head against the cabin door, to guard the apartment from being intruded upon, and then fall into a kind of slumber, dreaming that I was falling from a high precipice, bringing on the most torturing sensations, which made me start and spring to my feet the same as if I had been shot. I found my nervous system completely capsized; and the boys were continually telling me that the crew were only waiting a fitting opportunity to kill me if I went to rest, saying, "Oh! sir, they will have your life, and then kill us." "Oh! Captain Johnston, what will become of us?" I felt more for the boys than I did for myself—they would not for the world go forward to lend a hand to take in a sail or set one, unless I was on the deck, they seemed in such dread of the crew: the same young fellows who gave their oaths against me. I feel very sorry for one of them—he was the youngest. I had formed the strongest attachment to the boy, and intended to have him sent to school:—I was capable of teaching him navigation; but, poor little fellow, he gave me a helping hand down hill, and I hope the Lord will pardon him. In latitude 14 N., I sent up a new fore-topsail yard, and condemned the other, as it was sprung in two different places. The N.E. trades hung greatly to the eastward, and then had changed their position to the S.E.; in crossing them the ship would bear up N.N.E., N.E. by N. and N.E., with the yards well rounded in so as to carry a lower studding sail. After I got out of the tropics and near the parallel of 33, I fell in with very unsettled weather, such as I never saw before in those latitudes: the winds kept continually flying round the compass, accompanied with torrents of rain—I thought it would have brought on a hurricane, heaven and water appeared to be in one. From the darkness of the weather I anticipated something disagreeable, and made preparations accordingly: the small sails were all stowed, and orders given to take in the top-gallant sails, jib, and main-course, and to stand by and let go the topsail halyards: none of the people would lay aloft to haul these sails. The squall caught me, and walked off with the three top-gallant sails, the fore one brought the mast along with it and the top-mast head, although the yard was in the lifts; I could not get a soul to let go the mizzen topsail halyards, and consequently the mizzen topsail-yard was carried away:—160*l.* would not be equivalent to the damage sustained, without reckoning the detention on the voyage. I told the crew that if they did not exert themselves, I would send the ship to the bottom; this had a little weight with them, and they began to crawl about like so many bugs in a tar bucket. It was a good job for me that the squall did not last long; had it remained steadfast another hour, I should have been deprived of every stitch of canvas bent to the yards: I had all hands on deck that night until the wreck was cleared away, I think it was about the 20th of October, at 10 p.m., that I shortened sail, and hove to with the ship's head to the south-eastward; at daylight, made sail and run down along the coast of Pico with a pleasant breeze: at noon the winds were light. At 1 30 p.m. the town of Fayal hove in sight, when I hoisted my national colours, and kept the Island of Pico close aboard till I got nearly opposite Fayal. About 3 45, I observed the harbour-master approaching me in his boat; I immediately wore ship on the heel and braced sharp up on the larboard tack; I then ordered the boat to be manned with the ringleaders, C——, R——, L——, G——, J—— C——, Y——, S——, and a passenger-boy, named H. S——; the latter had requested to go on shore; and I, like a fool, granted his request, not thinking, of course, that my indulgence to the young fellow was to be converted into such base ingratitude—his passage to England had been given from charitable feelings, but he was under the guidance of a wicked mother. I took S—— in the boat, with the hope of falling in with an English doctor to examine his knee, and had him dressed up in my own clothes to make him a little decent on landing in a foreign part. This was the way in which I dressed and brought up these instruments who contributed so largely to my destruction, especially this fellow, who was the instrumental source of all the evils which happened on board of that ship. I ordered two pistols and my sword to be put in the boat, and when the harbour-master came within hail of the ship, I then shoved off—met him—and desired him to go on board and take charge of the ship until my return, and then started for the town. I think it was getting on for 4 30 when I got alongside of the landing-place; the Portuguese authorities were there, and questioned me in the usual form regarding the ship, from whence and where bound, &c.—the questions being satisfactorily answered I was then permitted to land: the Vice-Consul was standing there, and also Lloyd's agent—the latter presented me his card, and wished to know if he could render me any service; I thanked him, informed him that I had business with the Consul, and begged to be excused. I turned round to the boat's crew, and gave orders that they were not to leave the boat until my return, desiring Y—— to take charge of the pistols and sword; I then went to the Consulate, accompanied by the Vice-Consul, who introduced me to his senior. We were then ushered into a large chamber inclining to the right-hand of the great stair which I ascended from the street—the table in this apartment stood on the right-hand as I entered the door. After the customary compliments of the day were exchanged, I then related the circumstances of the mutiny; S—— detailed the whole account of it to his family. The Consul asked me if I thought there would be any doubt of their breaking out again; "for," said he, "if you feel at all doubtful, you can bring your ship to an anchor, take them on shore, and I will give them a hearing and send them home in irons—there are plenty of men here." He then detailed various cases similar to mine, and the trouble which he experienced with almost every vessel frequenting the port; he also mentioned a schooner not long gone from thence, and the great difficulty and annoyance he had experienced both from the crew and master of that craft. I then replied—"I believe you, sir, and have seen one or two instances of the same kind myself; but I have no accommodation for two crews, therefore I must chance them,—they have been quiet lately, and I will run the risk." I begged him to be as expeditious as possible in getting the stores on board, and can safely say that no man could have done it quicker. I was then introduced to his family circle, comprised of two young ladies and an elderly one, whom I took to be the governess: I was presented with a cup of tea, and entered into conversation with the eldest of the ladies—the two youngest were playing upon the piano-forte—a wonderful change for me to what I had to face every day. She told me how long she had been in the family, and several little domestic things quite unnecessary to mention here. During this time the passenger-boy came and informed me of the boat's crew having all gone on shore to a public-house and getting drunk; he said, "the man J—— is quite drunk." I communicated the intelligence to the Consul, stating that I would start and go in search of them. He said, "Do not feel the least alarmed, I will send after them and prevent them from getting adrift." I asked how long it would be before he could settle with me—he said, "not above a quarter of an hour; I am expecting the boat's return from the ship every minute." About half an hour after I was called upon by the Consul, to examine my account with him, amounting to 70*l*. or 80*l*. I signed it, took leave of his kind family, promising to bring them some Chinese curiosities on shore should I come to an anchor, and walked down in company with that gentleman to the boat. His men had been in search of my boat's crew, and were making towards the landing-place at the same time; they could be heard before they hove in sight; the man J—— was supported by two of his shipmates, and saluted me thus: "How does the ship bear?" I said, "Silence, sir, and jump into the boat," and asked the boy if he had my arms in safety. I then jumped into the boat, bidding the Consul good-bye, thanking him for his politeness, and shoved off. I must now interrupt the reader, and remind him of the charge of inebriation brought against me.

I walked direct to the Consulate in company with the Vice-consul, returned to the boat accompanied by the Consul, I touched nowhere going or coming, and drank nothing during my stay on shore; and if my demeanour, bearing, and station had not entitled me to the respect which the Consul's family were pleased to show me, I do not suppose they would have conferred their civilities upon me. The British Consul may deny these things, but I trust that he holds his character as a gentleman too high to call it untruth: he knows its authenticity to be as correct and true as there is a God in heaven.

J—— pulled the stroke-oar coming on shore; he was now too drunk to keep time or lay on his oar, but rather chose to be abusive both in speech and action. I ordered him to lay in his oar and lie down in the boat's bottom, but he refused to obey my order; I took the oar out of his hand and forced him to be still. I asked the crew what quantity of spirits they had drank, and the reply was, four glasses. I had a Chinese gong in the boat, and desired one of the boys to sound it, it was answered by the ship's bell, and a light was hoisted to show her position. She was about seven miles distant from the town. When I got alongside of the ship Johnson was dead asleep, I ordered them to send down tackle and hoist him out of the boat. This man had the privilege of giving his oath in preference to mine, and overturn his bestiality on my head. I then walked into the cabin and sent for the harbour-master; when he came, I produced my account with the Consul, wherein his own charge was included, and asked him which side of the Island he would recommend me to take. He said, "You had better adopt the S. E. side of Pico; if a breeze springs up the channel is dangerous between Pico and Fayal." I invited him to a glass of wine, gave his boat's crew some grog at his request, wished him good night, and parted. This differs materially from the evidence adduced. The crew stated that when I came on board I called the boy, and ordered my pistols, went to the gangway where M—— was employed taking in the stores, and commenced beating the man about the head in a ferocious and brutal manner. Now the lighter which conveyed the stores on board had arrived on shore, and brought me the receipt of the things having been duly received. No man would be so foolish as to sign a bill before seeing that he had received its value. My conduct on this occasion was neither that of a drunkard nor a lunatic. To attack a man in the execution of his duty, and in the presence of ten or twelve strangers, who were just returning to the town which I had just left, is an idea too absurd—these monstrosities sworn to have been committed by me, on the night I landed at Fayal, never existed, but form the most hateful and malicious piece of perjury ever suggested by the human heart. It is nothing else, because the contrary can be proved by living men. Had the truth been spoken between God and man, things would have had a very different aspect. Although credit may not in general be given to my statement, I still have the satisfaction of knowing that it will be *fully relied* on by those who thoroughly know me. I was the most free from drink of any man on board; there was neither lock nor key on my stores, and the boys had free access to them, plundering me right and left, dividing them with their most intimate cronies. When I had a gentlemanly officer with me these precautions were unnecessary, and when the unhappy change took place, I had made no provision to guard against it.

The watch was ordered below about half an hour after my return to the ship. M—— belonged to that watch, and relieved F—— at midnight. I walked the decks mostly all night—all quiet and asleep—the watch on deck picking out the softest plank and went soundly to rest. Nothing occurred, not a breath of wind. I got no sleep; I was months without a week's sleep, weeks without a day's sleep, days without an hour's sleep, and lastly I could not sleep at all. My eyes were the same as if they were placed into a bed of live ants. Daylight—the weather calm and hazy; the ship had only altered her position about five miles during the night.

I have now arrived at the most disastrous part of the voyage, and of the greatest import to me. I hope it will have some essential tendency in placing a clearer light upon one of the most brutal, barbarous, savage, and cruel acts ever perpetrated by the hand of mortal man. On the 22nd, at 8 a.m., I gave orders to erect stages over the starboard side of the ship, to commence painting, thinking it a pity to lose such a favourable opportunity; the sea smooth and the ship steady, and wishing to have her appearance a little ship-shape. I always took a pride in the ship I commanded, strained all my nerves in procuring abundance of fresh provisions &c. for the crew—I allowed them half a pint of wine each a day, from the time I had boarded the Frenchman. In the last port a live bullock was purchased, and sent on board in that condition expressly for their use, and fruit and vegetables to a large extent; these things are not customary, and I do not believe that any man in my situation could have surpassed my liberality to my crew. And I now give to the world the result of this day's proceedings, the violence which I committed, precisely as it happened, and as I hope to meet my Saviour at the day of judgment. The orders were given and treated with contempt. I inquired the reason of this unexpected and mutinous behaviour—their excuse to me was, "We are not to be ordered about by F——, who is no better than ourselves." I told them that he had my authority, and I expected them to obey him the same as if I had given the orders direct. I also reprimanded J—— for his drunken and improper conduct. I then directed the ship's head to Fayal, steered in that direction for a short space, with little blasts of wind, and at last came through the channel which separates the islands of Fayal and Pico. Seeing my disappointments multiplying, the crew witnessing my embarrassment, rejoicing at my failures and fruitless attempts to regain that port which I had just left, without complying with the Consul's advice, but trusting on their future behaviour. These things were intolerable, and, combined with my want of natural rest, drove me almost frantic, and only twelve miles from the land. I desired them to return to their duty. F—— lodged all the blame on M——, saying the fault lay with him—he also stated to M——s' face how unfaithfully he behaved to me during the conspiracy, and upbraided him for his infidelity on the night of the mutiny; he further stated, that M—— went direct from the cabin, on the evening of the 25th of September, after professing his friendship, and lent a hand to collect the weapons with which they intended to beat out my brains, and that this false man tried to smile his innocence down my throat; that B—— could prove it, and that when I was on shore he was forward with the crew, holding private consultations with them, and desiring the harbour-master to keep the ship farther from the land. B—— and T—— corroborated the first charge, F—— and S—— the latter. When I collected the crew, G—— was spokesman; I gave him three or four blows on the head with the hilt of the sword, and ordered them to be secured in irons. C—— stated that G—— threatened to take his life with a marlinspike if he refused to aid in killing me. I never struck him before nor after. I ordered the manacles on all the ring-leaders, with the exception of Thomas R——; he said he would return to his duty; I clapped him on the shoulder with my hand, and said, "That's a good fellow, Thomas." I then called M—— into my cabin, and said, "M——, I thank you and your friend for the misery and tribulation you have brought on me since I sailed from China—you are a false and deceitful fellow, but I am determined that you shall never deceive me from henceforth—I will take you home in irons—you have driven me to desperation." I caught him by the collar; he tried to escape me, but finding it difficult and above his strength, he sent his teeth through the heel of my right hand. I let go my hold, and reached my hand where the sword lay—he sprung to the cabin-door—I caught him a blow with the hilt over the head, which knocked off a piece of skin about the size of a sixpence. I did not see it until the following day. I ordered him to be put in irons. I did not order any shackle to be put round his neck; both cables were bent to the anchors. The charge was a falsity. The skin that I knocked off his head was the same that C—— and Y—— swore that I cut out with the sword in their presence—there was not a soul on board the ship who saw me.

At 6 p.m. the weather had a threatening appearance, with vivid flashes of lightning. I took in the small sails, and made other necessary preparations. Thomas R—— was knocking about the decks—he would neither work nor leave it alone, cursing and swearing, which made me think they must have brought spirits on board with them the night before. At eight the watch was sent below: R—— belonged to that watch, but remained on deck. I ordered those on deck to insist upon his going below. He persisted in remaining on deck, using all sorts of horrible threats. 8 30, Y—— and D—— came running into the cuddy, and called out, "Captain Johnston, R—— swears that he will take your life before 12 o'clock, and that you shall never see the light of another day." F—— had charge of the watch on deck: I desired him to send the man to me. He came, and I said, "Well, R——, you promised to be quiet; since then you have been trying everything in your power to provoke those who would be quiet but for your unruly ways. Is it because you are the bully and oracle of the ship that you are to frighten me? I tell you what it is, R——, if there were as many of you on board as there are chests of tea, I would defy you all to take my life; I have made a vow never to relinquish it without a desperate struggle." My feelings were now wrought up to the highest pitch—I had been harassed to the extreme, almost to death itself. I caught him by the collar, and made him spin round the cabin. I then put my hand on one of the bayonets lying on the cabin table, and said, "I have a mind to run you through." He caught the weapon by the centre, and a struggle ensued. He was a large and athletic man. The point of the bayonet came into contact with his left side, about three inches below the short rib; he then let go his grasp, and said, "I am stabbed!" "I hope not, Thomas," was my reply, and desired him to take a seat, and let me look at the part. He undid the waistband of his trousers, and showed me the wound. It was scarcely perceptible; not a drop of blood was to be seen. I said, "I don't think, Thomas, it is so dangerous as you imagine." He replied, "Oh yes, sir, I am beginning to feel quite sick." I took him in my arms, and laid him on a couch, and ordered the steward to fetch me half a glass of wine, which I gave to him. He drank it, and instantly became spasmodic. The spasms followed in rapid succession. I asked him to speak, but he made no reply. I then applied a little lint, dipped in balsam, to the part, and ordered him to be removed on deck, and sent for M——, and told him that I blamed him and his infernal gang for all this. The man was carried on deck, where he had fresh air. He again spoke, and sent B—— to me, requesting a little more wine, which was immediately granted. This happened about 9 p.m. At 10 I saw him lying on his mattress, and desired him to be conveyed to the top-gallant forecastle head, where the fresh air had free access to him. J—— promised to wait on him. There was not the least symptom of a scratch, nor a speck of blood on his body. This is a correct account respecting this man, and of the violence committed by me. It soon spread through the ship, that they murdered the man in order to criminate me. I do not mean to assert this charge against the crew, not having seen it. B. Y——, in giving his testimony on my trial, stated that I stabbed the man while he was sitting down, in three different parts of the body; and after calling M—— to behold the corpse, that I plunged the sword through his dead body. These gigantic enormities were never perpetrated by my hand. His evidence was of the most horrible and cruel description; his malignant feelings towards me were of the basest nature. Had I treated the boy badly or harshly during the voyage, I should not have felt the least surprised to see him perjure himself for the sake of revenge; but after writing home to his parents, saying that I behaved to him like a father, it made the thing appear to me dreadfully wicked. The same individual was the first who gave me notice of the plots forming against my life. He also denied having signed the entries in the log-book voluntarily, stating that I compelled him to do so by force, threatening to cut out his heart, to make ink of his blood—a very barbarous and unreasonable expression. These things are believed—the public have nothing else to go by but the testimony of my crew. After R—— was removed from the cabin, I then made preparations to blow the ship up, to frighten them. When these tidings spread through the crew, they all came flying to me, praying on their bended knees, and saying they would never disobey my orders, if I would only forbear, and try them once more.

On the 23rd, at 5 a.m., the tidings of R——'s death were conveyed to me by B——. At daylight, the winds moderate, and gloomy weather, with rainy appearance. At nine, I examined M—— and C——; they both confronted and accused each other. M—— charged C—— as being the instigator of the mutiny, and called him the demagogue of the ship's company. C——, in return, laid all the blame on M—— and R——, as being the first to propose to the crew to kill me. He furthermore pointed out the dates and nights on which they were carrying on their intrigues, and trying to inveigle the crew, till at last they consented. He finished by saying that M—— was as guilty as R——, and that he desired them to strike, when they were ordered to get the stages over to paint the ship. These things so exasperated me, that I did not know what to be about. I had often found M—— among the crew during the night, when he had charge of the watch, and no duty going forward, and I had often rebuked him for it. I now made use of threats—brandished the sword in his face—struck him several times over the body, with the flat of the weapon. I did not stab the man, as the witnesses stated: if the point or edge of that weapon had touched him at that moment, it would have gone right through the man. M—— said, "These —— murderous villains are making you believe that it is all my fault: take care they don't have your life before long." I then ordered him to be removed. There were no manacles on him at this time. In about three-quarters of an hour they brought another charge against him, just at the very time when I was in the highest state of excitement. I never sent for the man at stated intervals; I should never have thought of him, had it not been for the boys and one or two of the men, who kept me in hot water about their lives, representing him as being the sole cause of the last outbreak. This was the second time that S—— brought several new charges against him. On one occasion he attempted to pitch him overboard from the larboard cat head, and said, "By God, I will shoot you," seizing one of the pistols lying on the cabin table, loaded with ball, and presenting it to M——'s head. I saw him taking a deadly aim. M—— called out; I struck the weapon out of his hand with the sword with such violence, that it nearly severed one of S——'s toes from his foot. Had it gone off when S—— snapped it at M——, the man's brains would have been blown out, and as a matter of course laid to my charge. I ordered the man away. They requested to hang him. M—— said, "No, Captain Johnston, take me home and try me." These were the last words the man said to me: I never saw him afterwards. In about half an hour or so, the boy G—— came and told me that the crew had murdered M——, that they put a Spanish windlass round his body, and six of them hove on it, which made him call out, "Oh! you —— murderers, put it a little lower down, and finish me at once." Yes! D—— and S—— said that they stabbed him five or six times with their knives, C—— three times, S—— once; Y—— never touched him. So dreadfully did they crush the man's inside with the rope, that he vomited two large worms, which they exhibited round the deck, singing out, "We have taken two devils from him; there is no fear of his saying anything more against us." S—— was present when the man left, and also when the news of the murder was brought to me. I asked him what he thought of them? He said, "This is just the way that they would have served you and me, if you were not a man out of a thousand." "Well, carpenter," said I, "I will question them about it." I then sent for the principal persons: they came and acknowledged it, saying that it was a very good job, and that it was all his fault. Those who had no hand in the murder requested to be separated from the murderers. William B—— stated that his life was in danger, and that he was afraid to remain any longer with those who committed the crime. I desired the carpenter to allow this man to turn out and in with him, and live in the same cabin. The witnesses stated that I gave orders to kill M——, and instructed them how to do so; they likewise swore that I cut a piece out of M——'s head with a sword, whilst he was in irons. I have already related how the piece of skin was knocked off. My hand and arm had swollen to a great size, from the effect of the bite inflicted on that occasion. At 3 p.m. he was committed to the deep.

At eight the wind began to blow fresh, the topsails were double reefed, the jib and main-course stowed; they were all doing duty but the ringleaders, they were still in irons. I ordered the boys to go aloft and lend a hand to reef those sails; they said, "Oh! Captain Johnston, they will pitch us overboard." The young fellows would not move from my side. Does not this circumstance seem strange, that I was said to be cutting and carving the men to such an enormous extent from mere brutal gratification, and these boys would not stir from my side; they clung to me like so many leeches for protection. The idea of those clinging to a monster committing all the atrocities they speak of, well deserves investigation. It is quite apparent to every man, that if I had pursued the violence according to the interpretation of the evidence, that I must have appeared shockingly disgusting, and by no means fit for any human being to crave protection of. My explanation shall be short and simple on this subject:—On the evening of the 25th September, the witnesses said, "there was no mutiny on board; Captain Johnston was drunk, and went round the decks pricking the men in irons, cutting and wounding them violently;" they admitted that they took up arms against me, but their intentions were not to injure me, but merely to make me fast, to prevent me from killing them; the mate had given them the information that I was bent upon it. Capstan bars were not the things to make a man fast with. Another thing, they had the cabin doors to secure me below; I was only one man to twenty-eight. The charge of intoxication followed me to the island of Fayal, where I was said to have landed half gone in drink, and returned in a complete state of drunkenness. If these men were under such frightful apprehension for their lives, surely, whilst I was labouring under the influence, as they said, of drink, and demanding my arms before getting into the boat, this was the time for their putting themselves under the protection of the Consul, and to represent their dangerous situation: they ought to have done so if things were as they stated. Or they might, as the ship was close to the land, have taken to the boats, and would have done so, had they not been the guilty parties.

In the contest between me and Thos. R—— the evidence ran thus: "that I had been drinking all day, and quite sober at the time when I sent for him; took the sword, began to flourish it, hove it down; seized a bayonet, and stabbed him in three different parts." I never had the weapon in my hand. I have given the authentic account, just as it happened, and the cause of it.

The world cannot form the least conception how a lot of graceless and desperate set of wretches can worry a man until he becomes quite infuriated; these things have occurred to some of the most amiable men in society, where they have been bullied and trampled on till their lives have become burthensome and wretched. It was not a trifle that started me off; I had done my best to conciliate my crew; I had fulfilled a man's share of patience, perseverance, forbearance, endurance, and suffering, and am unconscious of having acted otherwise, and, in all probability, those who have had their philosophy bearing against me would have acted no better than myself.

I am now getting towards the close of my voyage. On the 25th it blew strong from the eastward, the ship under low sail; the three men in irons sent several messages supplicating to be released, promising to behave themselves with all due obedience and decorum. I was carrying taut sail on the ship, causing her to lurch heavily; they seemed to be much afraid, and I gave orders to enlarge them; but did not speak to any of them. They gave some account to the boys how they were enticed into the last outbreak. S—— brought me the log-book, and said that he had filled it up to yesterday at noon. I asked him if he had stated everything as it happened; he stated, "everything perfectly correct." I desired him to read it over, which he did. I found fault with the entries with regard to T. R——'s death; he said, "I never saw any contest between you; I saw him in fits, but did not know the cause of it." He then further said, "that all the parties who had signed stated it to be quite right; they had seen no dispute in the cabin." I then signed it. It was here where I criminated myself; my judgment was at fault, and my intellect clouded. If I had had the full possession of my intellectual faculties I should not have done so.

About the 1st of November, ship close-hauled, with the wind at E. N. E., the top-gallant sails stowed. I gave orders to set these sails when the watch was relieved at midnight, the weather having a fine appearance; the order to lay aft and set the main topgallant sail was passed along, as soon as they came on deck. Shortly after, the boy B——, and I think W. B——, came running aft, calling, "Captain Johnston, come sir, there is another mutiny, they will have F——'s life if you don't heave a hand, and come to his aid." I ran with all speed, took my arms along with me, met F—— and two of the crew bringing aft S. C—— and T. G——; they were both bleeding. I made inquiries how this originated. F—— stated that the watch were long in making their appearance, and that he walked forward to ascertain the reason, and these two fellows met him with their drawn knives in their hands, and were just about to plunge them into him, and he knocked them out of their hands, and that it was a good job his pistol missed fire, or they would have had the contents. I asked the two men who were there, if it was correct. They said, "Positively true;" took the lantern, searched for their knives, and found them on the spot where they stated, and brought them to me, when I found they were sharpened on both sides: I put them along with some weapons taken from the same men on a former occasion. I had them put under restraint again. I never spoke to them, nor committed the slightest violence.

3 and 4 a.m., I fell in with the experimental fleet, hove to, with their head to the southward. I hailed one of them in passing through the squadron. They answered me, but did not feel inclined to hold a parley. At daylight they made sail, and soon over-hauled me, and steered in the same direction. At 1 p.m. I signalized with the admiral. We were then in the chops of the Channel, the sea running too high to board him. In the evening the fleet hove to: I continued my course, and did not see them afterwards.

On the evening of the 6th, about 10 p.m., the Lizard light bearing by compass about N.N.E., blowing strong. Handed the top-gallant sails. Daylight—several ships in company. 11 a.m., the Plymouth pilot-boat boarded me, and requested to know if I wanted a man to pilot the ship to the Downs; I told him that I thought myself competent, and gave a short detail of the miseries and misfortunes of the voyage: wrote three letters—one to Lloyd's Committee, one to my owner, and one to my wife, wherein I enclosed a copy of the papers signed by the crew on the morning on which R—— committed suicide; also the heads of the mutiny, which caused the death of a seaman and the murder of M—— by the crew; begged of him to post the letters with all haste; delivered to him the ship's letter-bag; desired him to receive the postage for his trouble, which he very cordially acknowledged as being amply remunerative. He then shoved off, saying, "I think you had better come into Plymouth till the wind changes; there are several ships lying in this port wind-bound." I have ever regretted my error for not taking this man along with me at the low charge of 10*l.*; he would most assuredly have saved me from the last and horrible impeachment which was pending over me. About half an hour after he left me, I tacked ship to the S.S.W., cleared away the cabins, stowed the powder in the magazine, thinking all was right; I had kept it there with a light burning at my side, in readiness to blow her up, in the event of their making a brush upon me. The weather indicated westerly wind, ship breaking off by degrees as I stood to the southward.

This was the first day that I exchanged words with the woman B—— for I believe five months, and told her that I meant to give her in charge on my arrival, for urging my crew to revolt, and gave her to understand how she had unsexed herself on the voyage, and was an everlasting disgrace to womankind. I think this was all, and feel sorry I said so much—she made volumes of it, but it is of very little consequence. She was a woman of the coarsest character, abandoned to all the evil passions, knew nothing of the soft impulses of humanity which characterize the female character. She was immodest, without shame, destitute of womanly feeling, gloried in displaying her person, and in boasting of her impure connexions. She went direct to the crew and communicated what I had said to her, which made a little commotion among them. I think it was about 5 p.m. that I tacked ship to the eastward, she would head up nearly Channel course. I consulted the chart and gave orders to keep her a point and a half to windward of mid-channel course, in order to give the English coast a wide berth. About 8 30 p.m., I desired the boy to bring me a cup of tea; he had just placed it on the table, when bang went the cabin door, and out went the light—I received a heavy blow over the head, which felled me to the deck, a cut across the neck inflicted with a knife, and a bayonet-wound in the right leg. There were two passages to my private cabin, and I happened to be close to one of them; forced my way through; called the boy to fetch me a light; walked round by a different entry which led to the main cabin, and saw two or three moving off as fast as they could trudge. They had plunged the bayonets and their knives right through my bed and bedding, fancying that I had jumped into it for shelter. This was the reason why I gave them all in charge, feeling conscious they were all accessaries. When I presented myself in the cabin from which I had just retreated, I saw F—— striking A. N—— with my sword, I took the weapon out of his hand: there were three pistols lying in the stern windows, loaded with blank cartridges; they were discharged in the fray. I asked the man N—— how he came to be there; he said that D—— desired him to follow him aft. D—— called him a —— liar. I asked them all, and they all said that it was D—— who desired them. I then could clearly discern that D—— was the author of this most treacherous piece of villany. He was the woman B——'s chief bully; he was the greatest blackguard that ever trod a ship's plank, and a decided coward: he had professed to have been led into the first plot by his shipmates, and he now became the principal leader in the last, thinking to murder me under the vilest disguise. So long as I had kept the powder exposed and threatened to blow them up, they were kept more in check. They stated to the magistrate, on oath, that I sent for them, ordered them on their knees, and began to fire away and cut them severely with my sword: made J. M—— kneel down till I took a deadly aim, and discharged the contents of the weapon through the man's leg, and while I was taking the aim my hand dropped, which caused the shot to lodge in that part of the body. J. B—— and J. A—— were the only men who spoke near the truth; the persuasions of those who were thirsting after my blood had no impression on them. I never saw these two men in arms against my person, therefore it was very trivial what they had to say: 'tis true that they struck when the others did—I mean in refusing to do duty: they also asserted that the crew would have taken my life, were it not for the resistance I made in defending myself. There was every allowance shown to these two individuals, because their own lives were in absolute danger if they opposed the dispositions of their fellow companions. They both called on my family, and told them that I was as good a master as ever they sailed with. I lost two sails the same night, the evening of the 7th. On the 8th, at 4 p.m., I gave orders to tack ship; I was getting close to the Princess Charlotte shoal; she missed stays, and I found it absolutely necessary to wear her: I called out to the crew to lay aft, and stand by to round-in the after-braces; none of them would obey me, they got all stuck together. While the ship was drifting fast down to this dangerous shoal, I took hold of a broomstick lying close by my hand, and hove it amongst them; it caught J. A—— over the back—I did not intend it to strike the man, he being the same person which I have mentioned after sailing from Lambuck: the poor old fellow stated to the magistrate that I had struck him once in the Channel, but could not find out the date. On the 9th, about 9 a.m., the Deal pilot came on board, the South Foreland bearing by compass N.E. by E. At 2 p.m. I brought the ship to an anchor in Deal Roads, and gave the crew in charge of the Revenue Cutter; went on shore in his boat with the representative of the Liverpool house and Lloyd's agent; noted the protest, proceeded to the commander of the cutter's residence, gave him a minute account of the vexatious broils, and the miraculous way in which my life and the ship were preserved: went to the hotel, where I engaged a London pilot; made arrangements with him for the complement of men to work the ship to London; ordered some fresh beef to be sent on board; had a glass of sherry wine during the time I remained there; returned on board accompanied by the Liverpool agent, and found the ship in a disordered state. Those who were not in restraint began to annoy me. I told the chief officer of the cutter to send the people where they could not torment me, telling him that I only gave the men in his charge, and meant to retain my own commission until the ship was safely moored in London: this gentleman spoke very feelingly.

About 10, the pilot got the ship under weigh, and came to anchor at 2 a.m. At 4 the steam-tug came alongside, and I bargained with him to tow me to London; the anchor was again hove up, and I again started. After passing the Nore my consignee boarded me, in another steamer from London; congratulated me on my safe arrival; seemed much grieved and showed signs of sympathy; inquired if the log was filled up in due order, and made various inquiries about ship and cargo. 10th, at 4 p.m., came to a single anchor off Gravesend, the river pilot then took charge of the ship. At 9 30, weighed and proceeded—at 11 30, entered the West India Import Dock. On the 11th, at 8 a.m., I started for the city, and noted the protest a second time. About 1 p.m., I appeared at the police office, from thence I went to my lodgings—could not shut my eyes; in the morning medical aid was sent for; this gentleman asked me how long I had been without sleep, and if I felt pains in my head; I told him that I felt no pain but rather a pleasant sensation, till I laid my head down; it then had quite a different effect upon my nerves, which made me start the same as if something had struck me at the heart: he administered large doses of laudanum, but it did little or no good. On the 15th I was taken into custody, charged with wilful murder, and sent to Westminster prison. I now saw myself overwhelmed with everything calculated to degrade human nature—sunk to the lowest abyss of misery. I tried to make away with my life, but I was too sharply looked after, the watch came to my cell every quarter of an hour. The day after I was carried into the infirmary, placed under the charge of Dr. Lavies, the medical gentleman of the House of Correction; a man possessing a large heart glowing with the great soul of humanity: here I became insensible for a day or two; he also administered laudanum; he gave his evidence to the magistrate in these words—"I attended the captain when he came to prison, and found an apparently punctured wound in the back part of the right leg; it was healing, but inflammation had set in, which afterwards became extensive—it extended round the wound; there was extensive suppuration affecting the whole leg and thigh : he has been very ill—I suspect there was a bad habit of body, and there was besides much depression; there was no sign of delirium tremens, *nor any symptom to show that he had been recently addicted to drinking.*" This was on the day of my final examination, when I was committed for trial for the murder of W. R——, T. R——, and W. M——; also, for feloniously cutting and wounding S. C——, and several others of the ship's company. It seemed to me that they found it much easier to punish the innocent, instead of taking measures to reward the perpetrators of that fearful catastrophe in proportion to their merits.

On the 6th of February, 1846, I was tried, without having a soul to rebut the corrupt evidence adduced against me. The judge, in summing up, addressed the jury in these words: "I don't know what to make of this evidence; the only one we can depend upon is S——, and I shall read his over again." The jury then retired; and, after some deliberation, returned their verdict, "Not guilty, on the ground that the prisoner was then in a state of insanity." I was removed from the bar, without anything being said to me by the Court. On the 23rd I was conveyed to this institution (Bethlem Hospital), where I have been pronounced perfectly sane by the physicians; and I consider my imprisonment as a gross violation of the rights of the subject—a right which I have not forfeited. I was tried by an impartial judge and jury, who acquitted me on the ground of temporary insanity, and who ought to have been as much prejudiced against me as the public. They were called upon to decide my fate; and ought to be the proper persons to determine it, as it was left to their decision. If I am not to have the benefit of my verdict, I implore of the nation to have my trial over again, and let me have the right of the law. I am not a murderer—I never assassinated my fellow man. The violence which I committed was in defence of my life, and the property I had in charge; in accordance with the law of God, and the first law of nature. But although I was driven to a state of madness and desperation by my crew, I thank God that I am not an assassin.

I was not only considered a steady man, but moreover a temperate person, and an enemy to those who were addicted to drinking. I was also allowed to be a man of superior seamanship, by all ships' captains and ship-owners whom I served, and a thorough officer by all the seamen that sailed with me. I navigated that ship through the most intricate navigation in the world; run day and night without fear, and nothing happened of the slightest description. Always made the best of landfalls to a mile; went entirely by my observations of the heavenly bodies; never made computations from dead reckoning; never hove the log from the beginning of a voyage to the end. I had many years' practice, and was thought to be an expert lunarian, when I was chief officer of a ship. 'Tis true that my chronometers altered their rate; but this is no criterion to show that I was deficient in the art and science of navigation. I was taught the theory of navigation in one of the first academies in Edinburgh, where I likewise obtained a knowledge of mathematics, geometry, and nautical astronomy; was considered to have made great progress during my study, and passed muster as one well grounded in its first principles, from my former acquired knowledge. I never flogged man or boy; never witnessed such a thing in all my life; so disgusting and revolting has it always appeared to my feelings.

If I had been killed and pitched overboard, and the ship retaken, I should then have excited public sympathy, and been called a martyr. But, having braved the storm, and, after encountering difficulties unparalleled, brought the ship and its valuable cargo safely to port, I received, instead of sympathy, the indignation of the United Kingdom, who made no allowance for the circumstances in which I was placed.

In concluding, I feel it my duty to express my heartfelt gratitude to Mr. Cope, and the two medical gentlemen of Newgate, whose frankness, kindness, and generosity during my illness, can never be forgotten.

(Signed) G. JOHNSTON.

Appended to the printed statement issued by Captain Johnston, there are many valuable testimonials of character from persons of reputation and standing, written with the view of contradicting evidence by which it was attempted to prove, that the murders were committed when he was in a state of intoxication. But, though we may here say that, in our opinion, this charge is successfully rebutted, we pass this question by, as one beside the subject we have before us. Captain Johnston was, by a jury, declared to be not guilty, on the ground of insanity; and, therefore, the only question we have now to consider is his present condition of mind. Is he now labouring under mental disease; or is he at this moment a rational and responsible man? We have never seen Captain Johnston, neither have we had other means of forming an opinion of his state of mind, beyond that which is now in possession of the reader. It would, therefore, be very presumptuous in us, or any one else, to attempt to give a positive and decisive opinion, upon such evidence. But it is quite competent for us to say, that, from the evidence before us, there is a sufficiently strong presumption of recovery to warrant an inquiry into his state. From a review of the whole case, it appears to us to have been one of ordinary impulsive insanity, which, it is possible, may have been but of a limited and temporary character.*

There is no evidence of any predisposition to mental disease; neither is there any proof of a previous aberration of reason, to induce the supposition of confirmed insanity. While, on the other hand, from

* We have every reason to believe that the physicians of Bethlem Hospital have no hesitation in saying that Captain Johnston is not now insane.

the long-continued mental excitement he underwent, and the terror in which he was kept for many months during his homeward voyage, it is quite within the range of reason to believe, that he was overtaken by a sudden and irresistible invasion of mania, in which state he unconsciously acted; but from which, since the subsidence of the causes by which it was excited, he has providentially recovered. If this be so— if Captain Johnston be now in a sound condition of mind—is it not both unjust and unnecessary to detain him as a criminal lunatic, separating him from his family, and depriving him of the opportunity of engaging in any useful or profitable employment? If he be guilty of the murders with which he is charged, during a responsible state of mind, then justice requires another kind of punishment. But if he be not guilty, the same justice equally calls for his discharge. The only reason that can be assigned for his detention, is the continuation of the disease which led to his criminal conduct. This, however, should in no case be taken for granted; but from time to time the state of every patient should be inquired into, with a view either to his retention or discharge. We see no valid reason why a distinction should be drawn between cases of criminal insanity, and lunatics confined in ordinary asylums, or brought under the jurisdiction of the Court of Chancery. They all labour under the same affection, which manifests its presence in various ways: in one, by mischievous tendencies; in another, by suicidal symptoms; and, in a third, by vicious and criminal actions. And yet, while one class of lunatics may be—on evidence of recovery—discharged from confinement and from the control of the Court of Chancery, by the Lord Chancellor, or on the recommendation of the Commissioners, the other must submit—whether recovered or still insane—to a perpetual incarceration.

Surely if it is not considered prudent to give a criminal lunatic who may have recovered the use of his reason (such being the fact in the case of Captain Johnston) his unconditional liberation, justice demands—Christianity enforces—all the benevolent feelings of the heart dictate—the necessity of relaxing our present severe, harsh, and restricted system, and of permitting the unhappy prisoner some chance of obtaining his freedom. His liberation from confinement is quite compatible with the maintenance of the authority of the law, and with the safety and security of the public. Are we justified in dashing the cup of hope for ever from the lips of our poor afflicted fellow-creatures, thus closely confined within the dreary cells of Bethlem, and in the criminal wards of other public asylums?

Having broached this subject, we leave it now with confidence in the hands of a humane and justice-loving people; trusting that, ere long, the spirit of improvement which is brushing away the dust of ages from many a cherished institution, may find its way to the cells of Bethlem, and cast a beam of hope into the breasts of many who are languishing there in perpetual bondage. From a few words dropped by the Chancellor of the Exchequer in a recent speech in the House of Commons, we are led to hope that the whole question of the punishment of crime will ere long engage the attention of the legislature. Let the friends of humanity, then, not lose that opportunity to place the whole question of the treatment of criminal lunatics before Parliament, in such a way as to ensure attention to its claims, and we doubt not that success will follow the effort.

---

